# Evaluation of Small-Molecule Binding Site Prediction Methods on Membrane-Embedded Protein Interfaces

**DOI:** 10.1021/acs.jcim.5c00336

**Published:** 2025-07-02

**Authors:** Palina Pliushcheuskaya, Georg Künze

**Affiliations:** † Institute for Drug Discovery, Medical Faculty, University of Leipzig, Leipzig 04103, Germany; ‡ Interdisciplinary Center for Bioinformatics, University of Leipzig, Leipzig 04107, Germany; § Center for Scalable Data Analytics and Artificial Intelligence, University of Leipzig, Leipzig 04105, Germany

## Abstract

Increasing structural and biophysical evidence suggests that many drug molecules bind to the protein–membrane interface region in membrane protein structures. An important starting point for drug discovery is the determination of a ligand’s binding site; however, this information is missing for many membrane proteins, especially for their membrane-embedded parts. Therefore, we tested the performance of computational methods for ligand binding site prediction in the protein intramembrane region. We compiled data sets containing GPCR- and ion channel-ligand complexes and compared method performance relative to a soluble protein data set obtained from PDBBind. We tested state-of-the-art geometry-based (Fpocket, ConCavity), energy probe-based (FTSite), machine learning-based (P2Rank, GRaSP), and deep learning-based (PUResNet, DeepPocket, PUResNetV2.0) methods and evaluated them using the center-to-center distance (DCC) and discretized volume overlap (DVO) between the predicted binding site and the actual ligand position. The three best-ranking methods based on success rates on GPCRs were DeepPocket, PUResNetV2.0, and ConCavity, and for ion channels, these were DeepPocket, PUResNetV2.0, and FTSite. However, average DCC and DVO values were lower for all methods compared to the soluble protein data set, for which DVO and normalized DCC values ranked between 0.33 and 0.72 in their best case, respectively. In conclusion, this study provides an overview of the performance of state-of-the-art binding site prediction methods on their ability to identify pockets in the protein–membrane interface region. It also underscores the need for further method development in the prediction of protein–membrane ligand binding sites.

## Graphical abstract

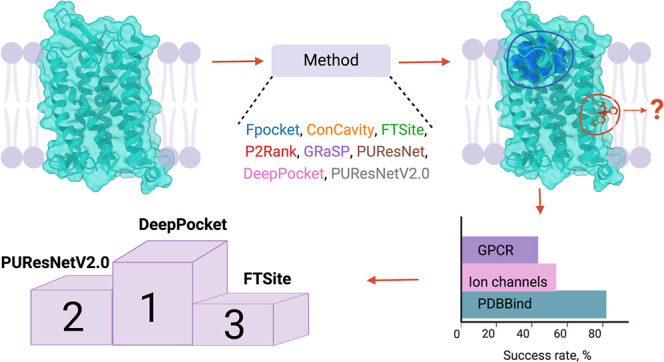

## Introduction

Many proteins convey their biological function by binding to other molecules including proteins, peptides, small organic molecules, or metal ions. Knowing the amino acid residues, which constitute the binding site of a protein, is essential for many applications, and the identification of small-molecule binding sites represents one of the first and necessary steps in structure-based drug discovery.^1,2^ Many computational drug discovery approaches, e.g., ligand docking, pharmacophore modeling, or virtual screening, require detailed knowledge of the druggable binding site(s) of a receptor protein.^3^ This is because, for a drug-like molecule to be pharmacologically active, it should have a high degree of structural and chemical complementarity to the protein surface, assuring favorable thermodynamic and kinetic binding properties. However, information about druggable binding sites is lacking for many proteins, hindering progress in drug discovery. Even in cases where small-molecule binding sites are known or can be reliably identified based on, e.g., protein surface shape or homology information, additional, difficult-to-detect binding sites may still be missed. In fact, proteins frequently possess more than one binding sites, which should be accurately analyzed regarding their potential to serve as drug target sites.^4,5^ An additional challenge lies in the identification of so-called cryptic binding sites, which are sites that are hidden in the unbound protein but become accessible after a conformational change. Cryptic pockets are of high interest for drug discovery because of their potential to target previously “undruggable” proteins.^6^ However, their detection can be difficult and requires techniques which study protein dynamics over time, like MD simulation^7,8^ and NMR spectroscopy,^9^ which are both time-consuming.

Several computational methods for predicting small-molecule binding sites starting with the unbound protein structure have been developed and applied, with the most recent ones showing improved performance by utilizing advances in machine learning and artificial intelligence.^10^ However, existing approaches focus mostly on soluble proteins or soluble protein domains because they represent the majority of protein–ligand complex structures in the Protein Databank (PDB).^11^ Thus, method development has mostly focused on soluble proteins, whereas prediction performance for other protein families, such as membrane proteins, has been less well documented. Nevertheless, binding site prediction for membrane proteins is of high relevance. This is demonstrated by the fact that the two largest groups of membrane proteins, G-protein coupled receptors (GPCRs) and ion channels, represent the targets for ca. 35% and 15% of currently used drugs on the market.^12^ Interestingly, an increasing number of structural and biophysical studies show that GPCRs and ion channels possess not only binding sites which are directly accessible from the aqueous environment but also binding sites that are located on their membrane-embedded surfaces.^13–15^ Binding of drugs to these membrane-embedded sites requires first permeation of the drug molecule into the membrane, followed by transport to its site of action at the protein–membrane interface. Interestingly, these sites can have allosteric and modulatory functions, and their discovery can potentially broaden the scope of drug discovery. One example is the P2Y_1_R purinergic receptor targeting drug BPTU (1-(2-(2-(*tert*-butyl)­phenoxy)­pyridin-3-yl)-3-(4-(trifluoromethoxy)­phenyl)­urea), which was developed by Bristol-Myers Squibb as a thrombosis inhibitor. BPTU was shown to bind completely on the outside of the 7-transmembrane domain of P2Y_1_R at a binding site formed by several hydrophobic residues.^13,16^ Further examples of ion channel drugs that target the lipid–protein interface region include retigabine, an antiepileptic drug, which activates KCNQ2 and KCNQ3 channels,^14,17^ dyclonine, an anesthetic and inhibitor of the TRPV3 channel,^15,18^ and zafirlukast, an antagonist of cysteinyl-leukotriene receptor with antiallergic function.^19,20^

Ideally, ligand binding site prediction methods should take different protein characteristics and environments into account. In the case of GPCRs and ion channels, the cell membrane represents an environment with special chemical and physical properties. For instance, the solvation energies and electrostatic energies of ligand molecules are different in the membrane than in water.^21–23^ Furthermore, the surrounding lipids influence the characteristics and accessibility of the binding sites, which may impede a compound from eliciting its effect. Molecules that bind to the intramembrane region tend to be more hydrophobic in their nature with higher log *P* values compared to soluble ligands, assuring sufficiently fast diffusion through the membrane to the target site. Increased hydrophobicity is attributed, e.g., to the incorporation of extended alkyl groups.^24,25^ For instance, the β_2_ adrenergic receptor agonist salbutamol showed three times lower affinity to treat asthma than the compound salmeterol. Although both share the same pharmacophoric group, salmeterol is a hydrophobic molecule, and compared to the hydrophilic nature of salbutamol, it effectively binds to the membrane, which leads to a long-lasting bronchi dilation.^26–29^ Another approach to increase membrane association is the introduction of basic amine groups. It was shown that amlodipine, a calcium channel blocker used to treat hypertension, is coordinated by membrane lipid molecules, which supports interaction with the ion channel fenestration site. The binding is attributed to the attraction between negatively charged lipid phosphate groups and the protonated ethanolamine group of amlodipine. Thereby, amlodipine exhibits a more long-lasting effect than other 1,4-dihydropyridines.^30^ However, it is worth noting that drugs targeting the membrane interface should maintain a proper balance between apolar and polar groups. Although drug transport through the membrane is facilitated by hydrophobic substituents, the larger part of a drug’s polar solvent accessible surface area (SASA) is buried within the protein binding site assisted by hydrogen bonds, and the smaller portion of polar SASA is exposed to membrane lipids.^29^ Nevertheless, comparison between intramembrane and water-exposed ligand binding sites shows that the former are more hydrophobic and contain a higher fraction of apolar and aromatic residues, which constitute ∼75% of intramembrane residues.^31^ These residues are more evenly distributed in intramembrane proteins, whereas in soluble domains, they tend to accumulate in the protein core. Aromatic residues are especially prominent at the protein–membrane interface, where they can facilitate cation–π interactions with positively charged head groups of lipid molecules. At the same time, charged and polar amino acids are less abundant in intramembrane regions.^32^ Moreover, ligand binding sites at the protein–membrane interface tend to be more flat and less concave than those found in globular soluble proteins,^24^ which makes their detection more challenging for, e.g., geometry-based binding site prediction methods. All of the points mentioned above highlight not only the relevance but also the difficulties associated with the identification of small-molecule binding sites in membrane proteins. Making progress in this area requires a systematic evaluation and comparison of the performance of different binding site prediction methods on membrane proteins with their binding sites located in the intramembrane region in order to establish the best practices for this group of proteins.

Binding site prediction methods are generally classified into geometry-based, energy-based, template-based, and machine learning- or deep learning-based methods.^10,33,34^ Aspects of the above methods may be combined in consensus approaches to combine advantages of the individual methodologies and boost their prediction performance.^35,36^ Geometry-based methods usually aim to find concave pockets on protein surfaces that will likely bind a potential ligand.^37^ SURFNET,^38^ LIGSITE,^39^ SiteFinder,^40^ and Fpocket^11^ belong to this group of prediction methods. Fpocket, which was already released in 2009, is still one of the state-of-the-art methods for pocket prediction.^41–43^ It is based on clustering of alpha spheres obtained by Voronoi tessellation of protein surfaces.^44^ Descriptors such as electronegativity of protein atoms, amino acid type, hydrophobicity, and number and polarity of alpha spheres are then used in partial least-squares regression to rank obtained pockets on how likely they can bind a small molecule. A list of differently sized pockets per protein structure is obtained, which can be further evaluated for their ability to bind a specific drug-like ligand. Advantages of Fpocket are its easy usage and fast computation time (1–3 s per structure).

Probe-based or energy-based methods rely on the exploration of protein surfaces with small organic probe molecules and calculation of the interaction energy between these probes and amino acid residues to estimate the binding ability of a potential ligand.^10^ Among energy-based methods are Q-SiteFinder,^45^ SiteHound,^46^ and FTSite.^47^ FTSite is the most recent energy-based binding site prediction method. It utilizes 16 different organic probe molecules, which mimic common building blocks of small organic molecules of various size, polarity, and shape.^48^ The determination of binding sites by FTSite involves the scanning of every protein surface point with probe molecules and calculation of their interaction energy using an empirical free energy function, clustering of probes based on their average free energy, and ranking of probe clusters by counting the number of nonbonded contacts between clusters and amino acid residues.^47^ Although FTSite achieves high accuracy without relying on any evolutionary input information, it is only available as a web server, requiring relatively long time to process a user submission (3–4 h for a protein of ∼350 amino acids).^49^

Template-based methods rely on detectable sequence and structural similarity between a query protein, for which ligand binding sites shall be determined, and a template protein, for which binding site(s) or cocrystallized ligand molecule(s) are known.^50^ After sequence alignment, ligand binding site information is transferred to the query protein.^10,51^ FINDSITE,^52^ 3DLigandSite,^53^ LIBRA,^54^ and bSiteFinder^55^ belong to this group of methods. While template-based methods are highly accurate, they depend on the availability of a homologous protein structure with a bound ligand. This information is not available for most proteins, limiting the application scope of template-based methods. Furthermore, this approach fails to predict hitherto unknown binding sites, limiting its use to only established drug targets.

An increasing number of binding site detection methods implement machine learning models, such as support vector machines (SVM), random forest, and Naïve Bayes classifier, for prediction.^56^ Here, ISMBLab-LIG,^57^ DeepSite,^58^ GRaSP,^59^ and P2Rank^60^ are the most prominent examples in this group. GRaSP is a supervised learning method based on the neighborhood graph theory,^59^ and P2Rank is a random forest classifier for predicting the ligandability of protein solvent accessible surface points.^60^ Furthermore, with the development of artificial intelligence, classical machine learning methods have been surpassed by deep learning approaches. Deep learning techniques make use of larger data sets and, opposite to the classical machine learning methods, which usually use handcrafted descriptors, can learn chemical features directly from the data set. Furthermore, deep learning models are more capable of learning complex patterns from data, but the way they arrive at predictions can be difficult to interpret. Usually utilizing convolutional neural networks (CNN), these methods showed a greater performance than statistical approaches with more successful interpretation of hidden relationships in the data.^56^ DeepSurf,^61^ kalasanty,^62^ PUResNet,^63^ PUResNetV2.0,^64^ DeepPocket,^65^ and many other deep learning methods have been developed to tackle pocket prediction tasks. DeepPocket implements a CNN scoring function to rescore pockets obtained from Fpocket to improve prediction accuracy.^65^ In PUResNet, a protein is treated as a 3D image, serving as input to a model derived from the principles of convolutional and residual neural networks, which outputs a 3D grid storing the voxelized probabilities that a voxel belongs to the binding cavity.^63^ PUResNetV2.0 was developed as an improved version of PUResNet by utilizing Minkowski CNN to account for the sparsity of the protein structure data set.^64^ It has to be noted that machine learning and deep learning-based methods require extensive data sets for training purposes and that their performance for systems that are hardly represented in the training set, such as membrane proteins, is insufficiently known.

Only a few systematic benchmarking studies have been conducted on this topic, but none of them looked specifically at membrane proteins. In a comparative assessment of geometry-based methods (i.e., SiteFinder,^40^ Fpocket,^11^ PocketFinder,^66^ and SiteMap^67^), Fpocket showed the overall highest accuracy.^68^ Another study evaluated 13 prediction methods, especially from the machine learning field (e.g., DeepPocket,^65^ PUResNet,^63^ IF-SitePred,^69^ etc.), which were compared to established methods like P2Rank^60,70^ or Fpocket.^11,71^ The study highlighted that rescoring Fpocket predictions with P2Rank and DeepPocket leads to more successful predictions. Because of the high relevance of identifying small-molecule ligand binding sites in the intramembrane region of GPCRs and ion channels, we analyzed and compared in this study the performance of several binding site prediction methods on this task. In our study, we tested the following methods (Table 1): Fpocket and FTSite from the group of geometry- and probe-based methods, respectively, GRaSP^59^ and P2Rank^60^ from the machine learning-based methods, as well as DeepPocket,^65^ PUResNet^63^ and PUResNetV2.0,^64^ belonging to the group of deep learning methods. In addition, we included one consensus method, ConCavity, which combines surface geometry-based and sequence-based approaches.^72^ Our results provide a detailed overview of the performance of these different methods and uncover possible directions for further method development in this area.

**Table 1:** Binding Site Prediction Methods Used in the Current Benchmarking Study

name	release year	type	availability
Fpocket^11^	2009	geometry-based	standalone application^79^
ConCavity^72^	2009	consensus (geometry + evolution)	standalone application^80^
FTSite^47^	2012	probe-based	web server^49^
P2Rank^60^	2018	machine learning	standalone application^81^
			web server^82^
GRaSP^59^	2020	machine learning	standalone application^83^
PUResNet^63^	2021	deep learning	standalone application^84^
DeepPocket^65^	2022	deep learning	standalone application^85^
PUResNetV2.0 ^64^	2024	deep learning	standalone application^86^
			web server^87^

## Methods

### Data Sets and Preprocessing

To assess the performance of binding site prediction methods on protein–membrane interface pockets, we compiled two membrane protein data sets. The first one contained GPCR structures taken from GPCR database (GPCRdb).^73,74^ We downloaded all structures that were bound to a small-molecule ligand and had a resolution of at least 3.0 Å. We analyzed the resulting structures and selected those that exhibited a drug-like ligand bound in the intramembrane region. We ensured that the GPCR structures downloaded from GPCRdb were already aligned to the membrane frame, in which the membrane bilayer normal is oriented parallel to the *Z*-axis, and its origin is at *Z* = 0 Å. The *Z*-coordinate of the ligand center-of-mass was measured to identify ligands located in the membrane bilayer within the range of −25 Å ≤ *Z* ≤ +25 Å. From the resulting set of structures, we only included those in the final data set, in which the ligand was bound on the outer, extrahelical surface but not in the canonical, intrahelical binding pocket. Other ligands, including lipids, sugars, and cofactors, were not further considered. The resulting GPCR data set contained 39 complexes (see Supporting Information Table S1).

A second test data set was compiled for ion channel structures. Complexes were taken from the mpstruc database.^75^ Ion channel proteins were filtered for the presence of drug-like ligands in the intramembrane region, as done before for GPCRs. This procedure yielded 59 ion channel–ligand complex structures (Table S2).

To also compare the performance of prediction methods between membrane proteins and soluble proteins, we prepared a third data set using soluble proteins from the PDBBind database (version 2019).^76,77^ Structures with a resolution of at least 3.0 Å and a 40% sequence similarity cutoff among each other were selected, yielding 420 protein–ligand complex structures for the third test data set (see Supporting Information Table S3).

All structures in the data sets were cleaned from nondrug-like ligands, e.g., lipids, water molecules, and ions using the clean_pdb.py script from the Rosetta software.^78^ Binding sites were defined as every amino acid residue within a 4.0 Å distance from the ligand. For analysis purposes, binding site residues and ligand residues were extracted from the PDB files. Distinction of the ligand-of-interest from nondrug-like ligands and determination of its intramembrane binding site location were done manually by structure visualization and crosschecking of the respective source publication. For multimeric proteins with the ligand-of-interest bound to each subunit, structures were separated into single chains and ligand extraction, and subsequent analyses were performed independently for each chain. In the cases of class C GPCRs, which form obligatory dimers, the entire dimeric structure was used as input to the calculation, and the binding site consisted of residues from both monomers.

### Binding Site Prediction Methods

Binding site prediction methods used in this benchmarking study are listed in Table 1 with their main characteristics. Additionally, we provide a more comprehensive overview of 45 binding site prediction methods found in the scientific literature in Supporting Information Table S4. Note that many of these methods are not available any longer or showed lower performance compared to the ones in Table 1 and, therefore, were not included.^68^ Template-based methods were also not included because web servers mentioned in the original papers were no longer available and because of the lack of template structures of GPCRs and ion channels, making these methods less suited for this task. In the end, we chose one method per category and more than one method from the machine learning and deep learning categories because of their potentially better performance. All of the chosen methods are freely available without restrictions.

Fpocket, GRaSP, P2Rank, PUResNet, DeepPocket, PUResNetV2.0, and ConCavity were installed on a local Linux computer utilizing their corresponding code repositories listed in Table 1. In case of FTSite, the web server, accessible from https://ftsite.bu.edu,^49^ was used to submit PDB files and make predictions. Every method outputs a ranked list of predicted pockets, which can be compared to the actual binding site, except for GRaSP, which predicts binding site residues. It assigns a binary label to every residue, where 1 means a residue is binding, and 0 means nonbinding. In order to have a consistent evaluation, the residues labeled as binding by GRaSP were clustered using the DBSCAN algorithm,^88^ followed by ranking each cluster using the propensity-for-ligand-binding (PLB) index by Soga et al.^89^ The PLB index considers the likelihood of each residue type to be found in ligand binding regions and sums these values up for the whole cluster. Thus, clusters with a higher PLB index are ranked more probable.

### Metrics and Evaluation

Two main metrics were used to evaluate the performance of each prediction method: “distance center to center” (DCC) and “discretized volume overlap” (DVO). DCC is the distance from the center of mass of the predicted binding site to the center of mass of the actual ligand binding site, calculated from the ligand coordinates in the protein–ligand PDB file. Predictions with DCC values of less than 4.0 Å were considered to be successful. DVO is the degree of overlap of the volumes of the predicted and actual binding pocket and is calculated using the Jaccard similarity coefficient formula

$$DVO=\frac{\left|Nr\cap Np\right|}{\left|Nr\cup Np\right|}$$

where Nr and Np are the number of points in the real and predicted binding pocket, respectively. Here, the DVO is defined as the number of points in the intersection of the real and predicted pocket divided by the number of points in their union. Predictions with DVO values of >0.2 were considered to be successful. In essence, points represent protein residue atoms with their 3D coordinates in the case of real binding sites. However, in the case of predictions, this can be either atoms, if a method outputs binding site residue assignments, or cloud points otherwise. Coordinates of atoms or cloud points were extracted from the respective PDB files utilizing PyBEL^90^ and NumPy^91^ python libraries. Fpocket, ConCavity, DeepPocket, PUResNet, and PUResNetV2.0 provide results in the PDB format; FTSite and P2Rank provide results in Pymol session files; and GRaSP provides a list of pocket residues in the CSV file format, which was converted into the PDB file format.

We evaluated the performance of each method based on the success rates they achieved in terms of DCC and DVO criteria considering the top N ranked pockets predicted by each method. Success rate is defined by the following formula

$$success\;rate=\frac{number\;of\;pockets\;with\;DCC\leq 4\;Å\;\left(DVO\geq 0.2\right)}{total\;number\;of\;pockets}$$

Success rates were calculated using the pockets ranked first by all methods (top-1 predictions) as well as the pockets ranked as first 3 (top-3), first 5 (top-5), and first 7 (top-7) predictions.

## Results and Discussion

### Location of Intramembrane Ligand Binding Sites at the Protein–Membrane Interface of GPCRs and Ion Channels

We compiled data sets of GPCR and ion channel structures, which contained ligands located in the membrane bilayer region and bound to the outer protein–membrane interface in these structures. The intrahelical orthosteric binding pocket in GPCRs and the central pore of ion channels were not included, as they are directly accessible from the aqueous phase and represent easy-to-detect binding sites. All structures were aligned to the membrane frame, in which the membrane bilayer is oriented along the *Z*-axis, and its center is located at *Z* = 0 Å.^92^ This allowed comparison of the ligand positions in a common reference frame and with binding site positions reported earlier for GPCRs.^93^ Following the pocket location annotation procedure described by Peter et al.,^93^ each GPCR ligand was assigned one of 19 possible extrahelical binding sites. Figure 1 shows the location of these extrahelical binding sites mapped on a GPCR structure with one representative ligand per binding site.

**Figure 1:**
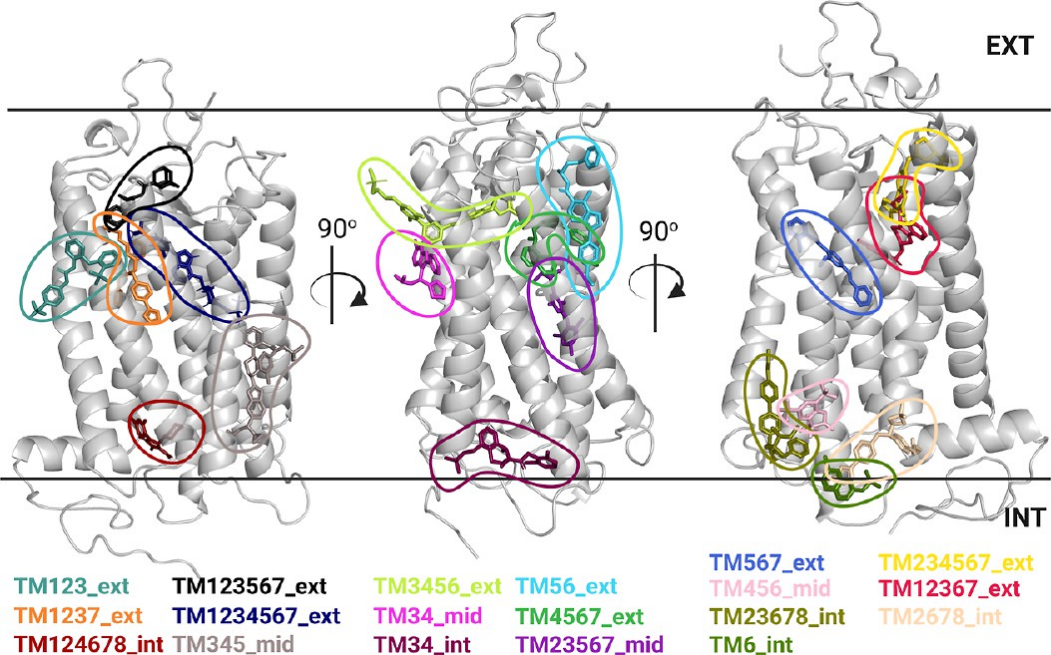
Extrahelical binding sites of GPCR structures analyzed by different prediction methods in this study. For each binding site, one representative ligand from the data set is depicted. The definition and names of the binding sites were taken from.^93^ TM—transmembrane helix, ext—exterior, int—interior, mid—middle sites. Digits represent numbers of GPCR transmembrane helices. Black solid lines represent the boundaries of the membrane bilayer. EXT—extracellular side, INT—intracellular side. The structure of CC chemokine receptor 2 (PDB code: 5T1A)^94^ was used to highlight different ligand sites. Ligand names and corresponding PDB codes that were used to represent binding sites can be found in Supporting Information Table S5.

In the same way, we aligned all ion channel structures to a common reference frame in the membrane in order to determine common binding site locations. Figure 2 exemplary highlights intramembrane ligand binding sites in representative structures of the transient receptor potential vanilloid 5 (TRPV5) channel (Figure 2A), two pore domain potassium ion channel TREK2 (Figure 2B), and voltage-gated potassium ion channel KCNQ2 (Figure 2C). Tables listing all structures in the GPCR and ion channel data sets are available in the Supporting Information (Tables S1 and S2).

**Figure 2:**
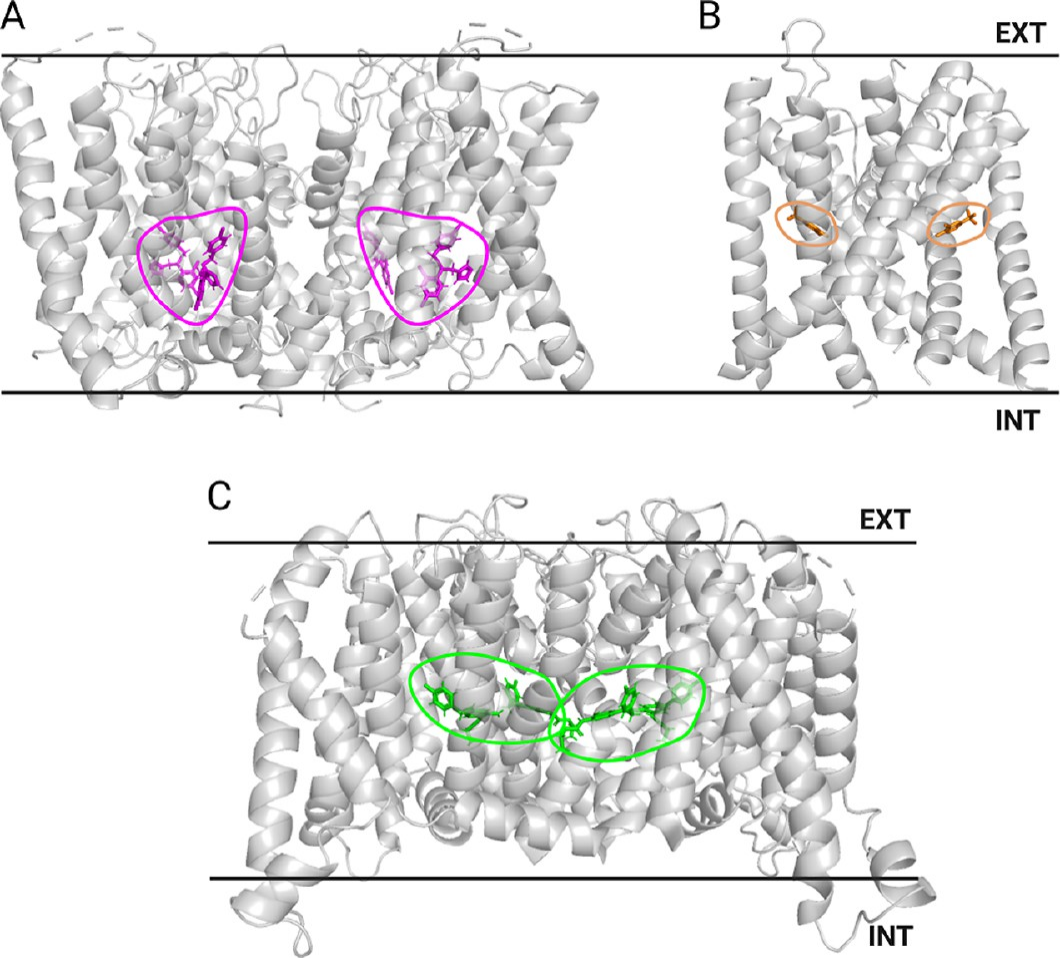
Examples of intramembrane binding sites and their respective ligands for three different ion channels used in this study. (A) Transient receptor potential vanilloid 5 (TRPV5) channel with econazole (PDB: 6B5V),^95^ (B) two pore domain potassium ion channel TREK2 with a brominated fluoxetine derivative (PDB: 4XDL),^96^ and (C) KCNQ2 potassium channel with retigabine (PDB: 7CR7).^14^ Only the membrane-embedded parts of the three ion channels are shown to better visualize the small molecule ligands. Black solid lines represent the boundaries of the membrane bilayer. EXT—extracellular side, INT—intracellular side.

To further analyze the spatial distribution of ligand binding sites, their positions were projected onto the membrane normal (i.e., *Z*-axis). Figure 3A shows binding sites on the extrahelical GPCR surface range from *Z* = −24 Å, in the lower membrane leaflet, to *Z* = +20 Å, in the upper membrane leaflet. Colors of the GPCR binding sites correspond to those used in Figure 1 for the representative ligand structures.

**Figure 3:**
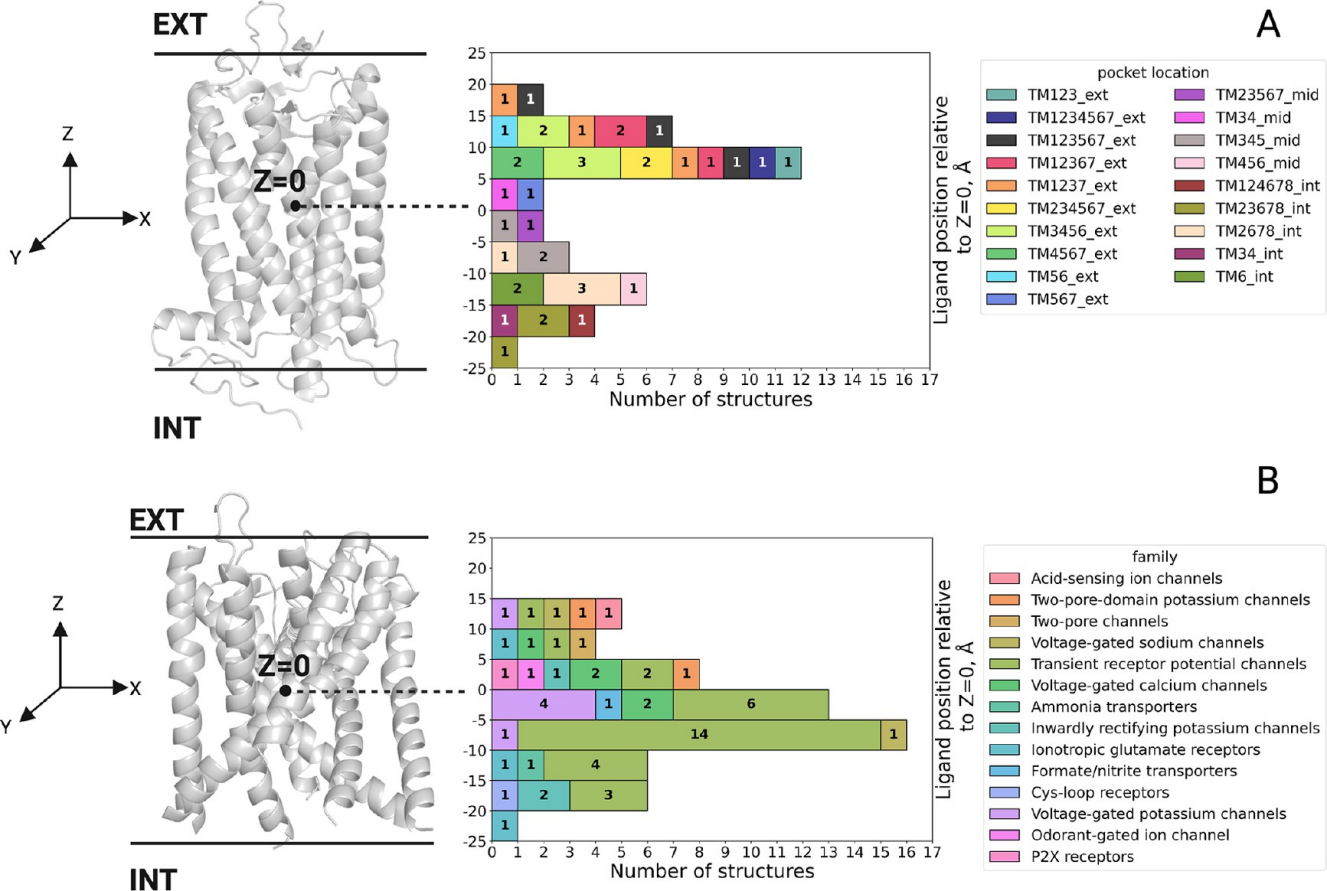
Location of ligand binding sites at the protein–membrane interface region. The number of binding sites along the membrane *Z*-axis is shown for (A) GPCRs and (B) ion channels. *Z* = 0 represents the bilayer center, positive values indicate a position above the membrane center, and negative values below the center. Black solid lines represent the boundaries of the membrane bilayer. EXT—extracellular side, INT—intracellular side. The colors and labels in (A) indicate the different binding site classifications defined in ref 93 and used in Figure 1. TM—transmembrane helix, ext—exterior, int—interior, mid—middle sites. Digits represent numbers of GPCR transmembrane helices. A representative GPCR structure is shown on the left side. The colors in (B) indicate the different ion channel families in the data set. A representative ion channel structure is shown on the left with only the membrane-embedded region highlighted.

Additionally, we analyzed the distribution of intramembrane ligand binding sites in our ion channel structure data set in terms of their *Z*-coordinate across the membrane. Figure 3B differentiates binding sites by their origin from different ion channel families. It can be seen in Figure 3B that membrane interface binding sites in ion channel structures range from *Z* = −22 Å in the lower leaflet to *Z* = +14 Å above the membrane center. The largest number of ligands bound at the protein–membrane interface region was found for transient receptor potential (TRP) channels, which reflects their widespread participation in various biological processes and the fact that TRP channels have been widely studied as important pharmacological targets.^97^

### Properties of Intramembrane Binding Sites in GPCRs and Ion Channels Differ from Those of Soluble Protein Binding Sites

In the next step, we analyzed the binding sites in our data sets regarding their amino acid composition and evolutionary conservation. We divided the amino acid residues in five groups (hydrophobic, aromatic, charged, polar, other) and calculated the fraction of each group and each amino acid type across our GPCR, ion channel, and PDBBind data sets (Figure 4).

**Figure 4:**
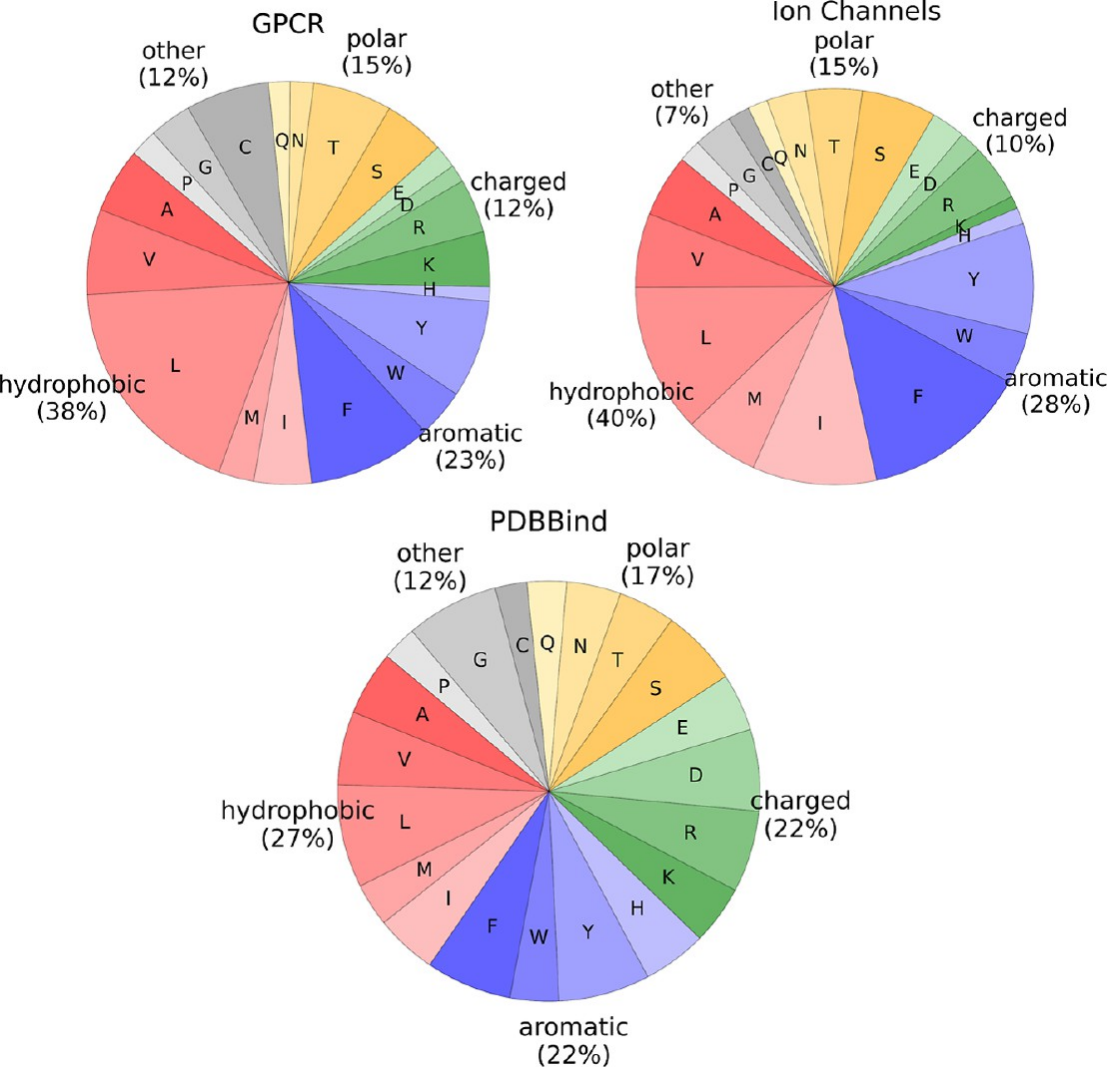
Average amino acid type composition of the binding sites for the three data sets used in this study. The fraction of hydrophobic, aromatic, polar, charged, and other amino acids is stated.

As illustrated in Figure 4, binding sites in GPCR and ion channel structures contain more hydrophobic amino acids compared to the ones in soluble proteins from the PDBBind data set (38% for GPCR and 40% for ion channel data sets, compared to 27% for PDBBind). Aromatic residues are a bit more often found in intramembrane binding sites, albeit not to a large extent (23% and 28% for GPCR and ion channel structures, respectively, compared to 22% for PDBBind). By contrast, they contain a lower number of charged residues compared to soluble protein binding pockets (12% and 10% for GPCR and ion channels, respectively, compared to 22% for PDBBind). Furthermore, polar residues are also less common in GPCR and ion channel binding sites (both 15%) compared to those in proteins from PDBBind (17%). In conclusion, this simple comparison highlights the significant physicochemical differences between pockets in soluble and membrane proteins and motivated us to further analyze the performance of binding site prediction methods on these two groups.

Another relevant difference between binding sites in membrane proteins and those in soluble proteins, which may affect prediction accuracy, could be the level of amino acid evolutionary conservation, which is an input feature used by several methods. Thus, we calculated conservation scores for all residues in all three data sets utilizing the ConSurf Web server.^98,99^ We focused on the surface-exposed binding site residues and compared the average conservation scores of the surface-exposed binding site residues to all other nonbinding site residues in PDBBind data set. In GPCR and ion channel data sets, we additionally extracted the intramembrane region and compared conservation scores of ligand binding site residues to those of all other residues at the protein–membrane interface (−25 Å ≤ *Z* ≤ +25 Å). The ConSurf method assigns conservation scores from 1 to 9, with higher scores meaning higher conservation levels.

Conservation scores of residues at the protein–membrane interface for the proteins in our GPCR and ion channel data sets show a broad distribution with the average conservation score around 6–7 (Figure 5B,C). For the analyzed GPCR structures, conservation scores of the surface binding site residues are on average one order higher than the scores of all other intramembrane surface residues. Intramembrane binding site residues have a mean conservation score of 6 compared to 5 for all other intramembrane surface residues. A similar trend is observed for the ion channel structures in our data set: binding site residues have slightly higher conservation scores than nonbinding site surface residues. The average conservation score for the analyzed ion channel ligand binding sites is 7 compared to an average score of 6 for all other surface-exposed and intramembrane residues. Nevertheless, residue conservation scores between binding sites and other intramembrane residues are quite similar, which can create challenges in the pocket prediction. For instance, despite having comparable evolutionary scores, extrahelical binding sites in GPCRs may be considered less “ligandable” when compared to other intrahelical pockets due to other properties, which render extrahelical sites less preferable, e.g., a low surface concavity or a low fraction of hydrogen-bond forming residues, etc. In comparison, binding residues in soluble proteins exhibit much higher mean conservation score compared to those of all other nonbinding residues (score of 8 to that of 5) (Figure 5A). This makes binding sites more easily detectable when applying binding site prediction methods that utilize evolutionary data, whereas these intricacies in intramembrane interface binding sites emphasize the need for pocket prediction methods that will consider the distinct characters of the membrane environment.

**Figure 5:**
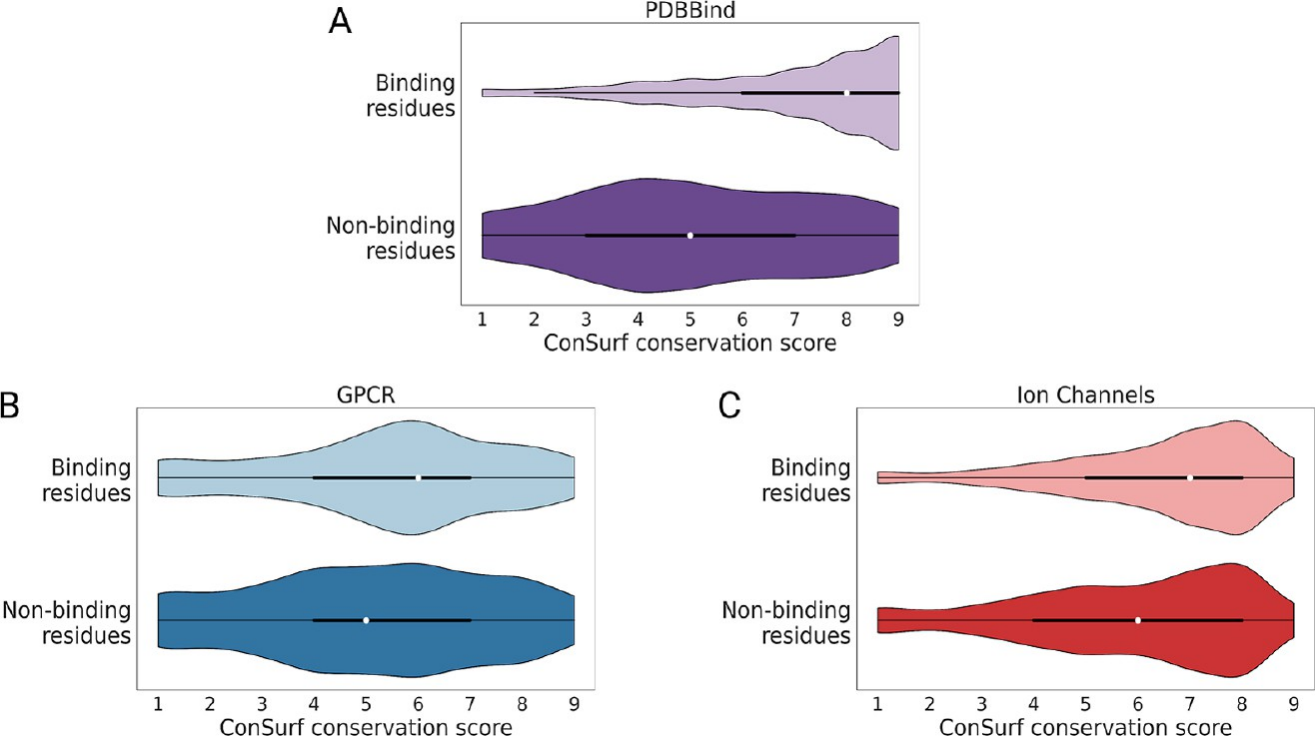
Conservation scores of binding site versus nonbinding site residues. All residues located on the protein surface in (A) PDBBind and additionally in the membrane bilayer region of (B) GPCRs and (C) ion channels were considered and divided into binding site and nonbinding site residues. Scores were calculated using ConSurf.^98,99^ Scores of 1 represent less conserved residues, and scores of 9 represent highly conserved residues.

In addition, we calculated several physicochemical properties for the ligand molecules in the GPCR, ion channel, and PDBBind data sets and compared their distribution. The properties, which showed the most pronounced differences, are highlighted in Figure 6.

**Figure 6:**
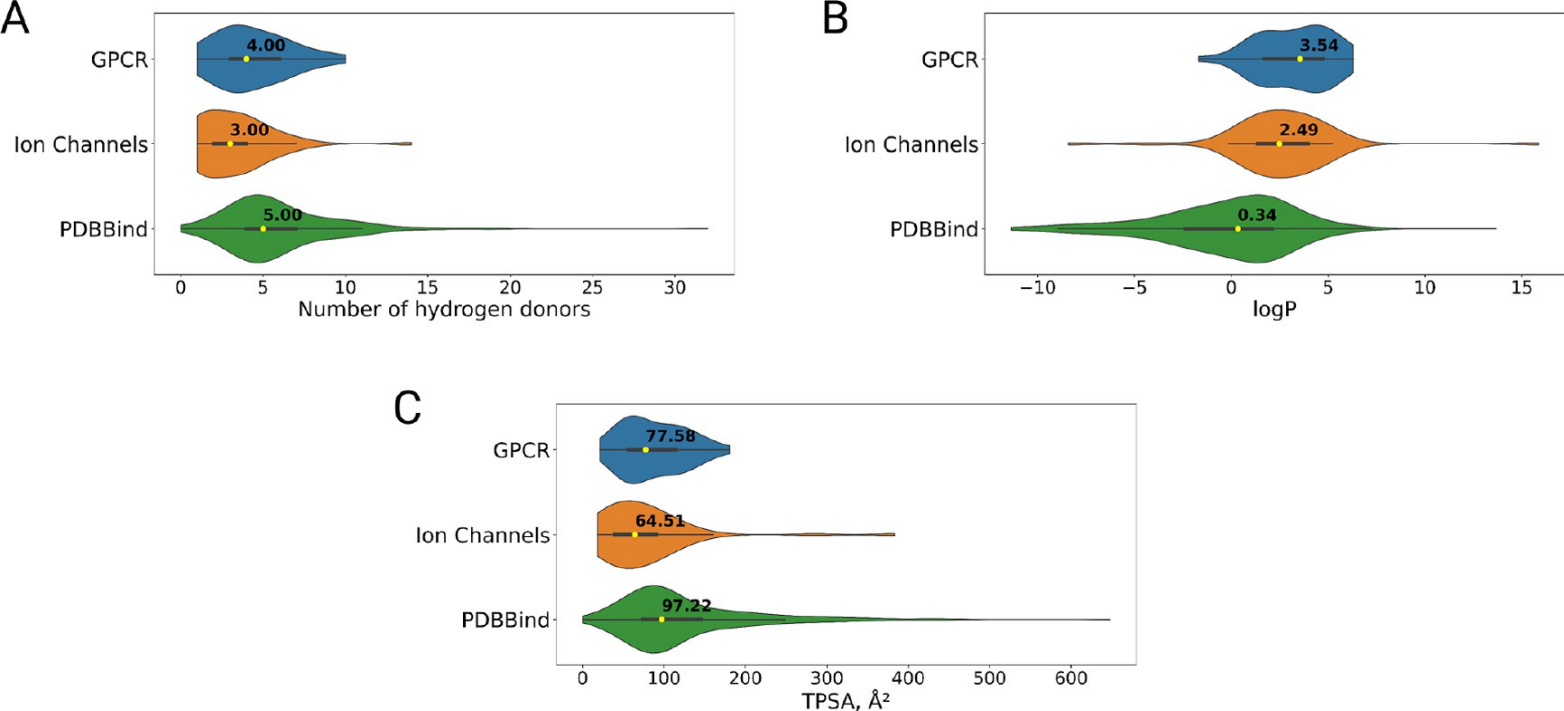
Distributions of ligand properties in GPCR, ion channel, and PDBBind data sets. (A) Number of hydrogen donors. (B) Octanol–water partition coefficient (log *P*). (C) Topological polar surface area (TPSA).

Consistent with the more hydrophobic character of the binding sites in the GPCR and ion channel data sets (Figure 4), their ligands display a higher value of the octanol–water partition coefficient (log *P*) and a lower TPSA. Furthermore, the number of hydrogen bond donors (HBDs) is lower compared to ligands in the PDBBind data set. The average log *P* values were 3.54 and 2.49 for GPCR and ion channel ligands, respectively, showing their preference toward a more lipophilic environment. In PDBBind, the mean log *P* value was calculated to be 0.34 in accordance with the higher number of polar and charged binding site residues in soluble proteins (Figure 6B). For the number of HBDs and TPSA, an opposite trend was found. On average there are 5 HBDs in PDBBind’s ligands, but only 4 and 3 in the GPCR and ion channel ligands, respectively (Figure 6A). TPSA is also highest in PDBBind, achieving ∼97 Å^2^, and much less in GPCR and ion channel ligands (78 and 65 Å^2^, respectively) (Figure 6C). This showcases the physicochemical differences between ligands that are prone to bind to more hydrophobic pockets found in the intramembrane protein regions and their soluble counterparts.

### Current Binding Site Prediction Methods Have Lower Success Rates on Protein–Membrane Interface Sites than on Soluble Protein Binding Sites

Next, we conducted an extensive benchmark of the binding site prediction methods for their capability to predict pockets located in the intramembrane region. We calculated DCC and DVO metrics across every obtained pocket prediction for each of the proteins in the three data sets. As described in the Methods, pockets that pass DCC ≤ 4 Å and DVO ≥ 0.2 criteria were considered correctly predicted. The success rate of correct pocket predictions was calculated for the top one, three, five, and seven ranked pockets. In Figure 7, the success rates obtained by each method on the GPCR, ion channel, or PDBBind data sets, respectively, are compared. It can be clearly seen that all methods perform best on the PDBBind data set containing only soluble proteins, both according to DCC as well as DVO metrics. Additional side-by-side comparisons of the top-1 and top-7 success rates for every method across all data sets can be seen in Supporting Information Figure S1, illustrating that the best performance in all cases is obtained for the PDBBind data set. In general, success rates based on DVO are higher than success rates based on DCC. This can be attributed to the choice of the DVO cutoff value of 20% pocket volume overlap. There exists no clear recommendation for the DVO cutoff value in the scientific literature, which is opposite to the DCC metric, for which 4 Å is the established cutoff value.^11,47,100,101^ For consistent interpretations, we will compare, in the following, the success rates obtained using the top-7 pocket predictions of every method.

**Figure 7:**
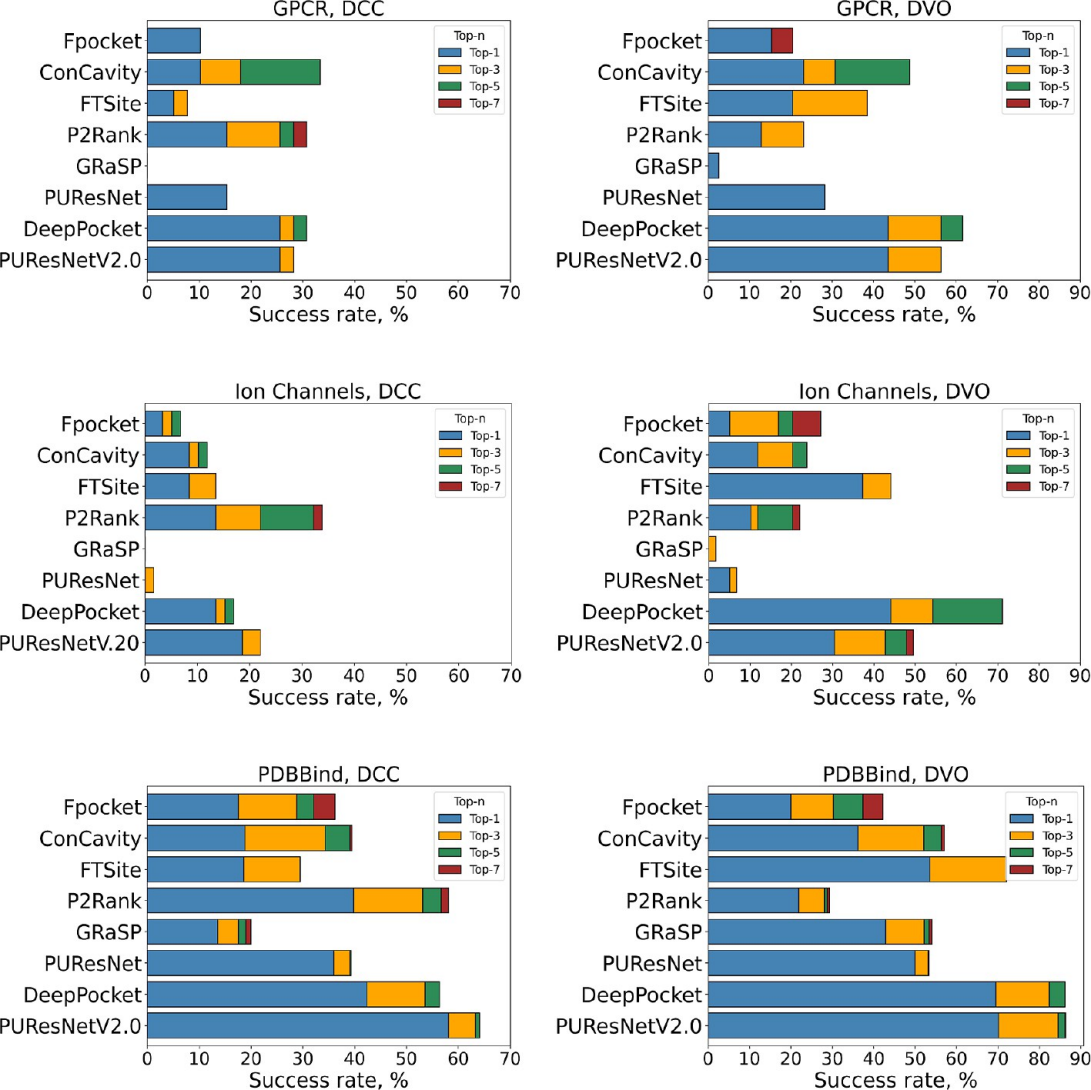
Performance comparison of ligand binding site prediction methods tested on GPCR, ion channel, and PBDBind data sets, respectively. The left-hand side plots show the success rate according to DCC, and the right-hand side plots compare the success rate according to DVO. *X*-axes represent top-n success rates with top-n being top-1, top-3, top-5, and top-7.

The deep learning-based DeepPocket method outperforms all other methods in almost every case across all data sets. The success rate of DeepPocket in terms of top-7 DCC is slightly higher for the GPCR data set (31%) than for the ion channel data set (17%), whereas the top-7 DVO success rates are comparable (62% on GPCRs and 71% on ion channels). By contrast, DeepPocket achieved even higher success rates for the PDBBind data set of 56% and 86% in the top-7 DCC and DVO, respectively.

The second best-performing method was PUResNetV2.0, which is another recently developed deep learning-based method. For the GPCR data set, PUResNetV2.0 achieved success rates of 28% and 56% in the top-7 DCC and top-7 DVO categories, respectively. Ion channels were predicted with 22% and 50% success rates, according to DCC and DVO, respectively. However, the success rates accomplished for the PDBBind data set were still higher than those for GPCRs and ion channels (64% and 86% in top-7 DCC and DVO, respectively).

P2Rank, which is a random forest classifier, managed to predict binding sites in GPCRs and ion channels also fairly well and achieved good performance using the top-7 DCC metric. It accomplished success rates of 31% and 34% on GPCRs and ion channels, respectively. These numbers are still almost twice as low compared to those of PDBBind, where P2Rank predicted 58% of binding sites correctly using the top-7 DCC metric. However, according to DVO, P2Rank ranked on the sixth place, both for ion channels and GPCRs. The top-7 DVO success rate reached 23% in GPCRs and 22% in ion channels, which is comparable to the result obtained for the PDBBind data set, where P2Rank was only able to correctly predict 29% of binding sites according to top-7 DVO.

PUResNet, the precursor method of PUResNetV2.0, failed to correctly predict ion channel intramembrane binding sites, where it achieved only the second lowest success rates (2% and 7% according to top-7 DCC and DVO, respectively). For the GPCR data set, PUResNet was ranked on the fifth place and achieved success rates of 15% and 28% according to DCC and DVO, which was still considerably lower than the success rates accomplished for the PDBBind data set (39% and 53% in top-7 DCC and top-7 DVO).

FTSite showed higher success rates using the top-7 DVO metric, achieving 38%, 44%, and 72% on GPCR, ion channel, and PDBBind data sets, respectively. It was the third best method after DeepPocket and PUResNetV2.0 in this category. In contrast, the performance by the DCC metric was considerably worse. Binding sites in GPCRs and ion channels were correctly predicted with only 8% and 14% success rates, respectively. For PDBBind, FTSite prediction resulted in 30% success rate in the top-7 DCC category.

ConCavity was the third best method on the GPCR data set, after DeepPocket and PUResNetV2.0, when considering the top-7 DVO metric (49% success rate) and the best method according to top-7 DCC (33% success rate), although with only a small margin to the next best methods DeepPocket and P2Rank. The success rate for the ion channel data set was again considerably lower (12% and 24% in top-7 DCC and top-7 DVO, respectively). In this case, ConCavity achieved the fifth place, the same as for the PDBBind data set (40% and 57% in top-7 DCC and DVO, respectively).

Fpocket achieved success rates of 21% and 27% for GPCRs and ion channels, respectively, according to top-7 DVO, but in terms of DCC, the success rates were lower (10% and 7% for GPCRs and ion channels, respectively). In a similar way, Fpocket ranked only the sixth place using the PDBBind data set, however, with overall higher success rates compared to membrane proteins (36% and 42% in DCC and DVO, respectively).

Finally, the GRaSP method had the lowest performance among all methods tested and failed to predict almost any intramembrane ligand binding pocket correctly. The top-7 DCC and DVO values were consistently below 10% (for GPCRs, 0% and 3% for DCC and DVO, respectively, and for ion channels, 0% and 2%, respectively). When applied to soluble proteins, GRaSP was also one of the worst performing methods with only 20% success rate when considering top-7 DCC, while it was slightly better in terms of DVO (54% success rate).

Some of the trends in the method performance observed above may be directly related to how individual methods were designed and trained. For instance, in the training of DeepPocket, the detection of protein curvature was emphasized, which may explain why its performance gain compared to the other methods is most prominent when assessed by the DVO metric as opposed to DCC. Furthermore, the low performance of PUResNet on the ion channel data set is probably caused by the fact that PUResNet is not able to predict binding sites in structures containing multiple subunits and the fact that ion channels are often multimers, with ligand binding sites shared between multiple subunits. In contrast, the majority of GPCR structures in our data set and proteins in PDBBind are monomeric, explaining the better performance of PUResNet for these data sets. This limitation of PUResNet was corrected in its successor PUResNetV2.0, which clearly showed better performance.

P2Rank is among the three best-performing methods according to DCC but performed considerably worse when looking at DVO. A possible explanation for this is an underestimation of the extent of volume overlap between the predicted and real binding pockets by P2Rank. Since the output of the method is a set of solvent accessible points and not a set of protein residues, predicted pockets tend to have smaller volumes and less pronounced shapes, leading also to smaller pocket overlaps.

The overall medium to low performance of Fpocket could be partially due to the fact that it was trained to detect any type of binding sites, not only of drug-like molecules but also of other types of molecules. This might have produced lower success rates because the druggable binding sites might have been ranked as not the top predictions. A possible explanation for the significantly worse results obtained with GRaSP across all data sets is the variability in the clustering of binding residues. While GRaSP classifies residues as either binding or nonbinding, it fails to group them into spatially distinct binding site clusters and to score these clusters. We employed DBSCAN to cluster positive residues, using a maximum distance of 8 Å between two residues in one cluster, which is a commonly used cutoff value.^102^ Further fine-tuning of the cutoff could potentially improve the results for GRaSP but is beyond the scope of this study.

It is worth noting that the high success rates on our PDBBind data set, which were achieved with the PUResNetV2.0 method, could be attributed to the fact that half of the structures were also present in the training set, which was used for PUResNetV2.0 development. Nevertheless, in the case of all the other prediction methods, there was no overlap between our data sets and the ones previously used for method training.

### Distribution of DCC and DVO Values Shows that PUResNetV2.0, DeepPocket, and FTSite Achieved the Overall Best Predictions for Membrane Proteins

In addition to analyzing success rates achieved by the individual methods, we also analyzed the distribution of DCC and DVO values of the top-1 predictions across all data sets. In general, DCC values tend to be high and could reach ∼30–40 Å. For better visual comparison of DCC value distributions across the different methods and data sets and with respect to DVO value distributions, we show normalized DCC values in Figure 8. DCC values were normalized to fall in the range between 0 and 1 by taking the exponential function of the negative DCC value divided by ten. Figure 8 shows violin plots of DCC and DVO value distributions produced by each method for the three data sets. The higher the median of these distributions, the better the performance of a particular method.

**Figure 8:**
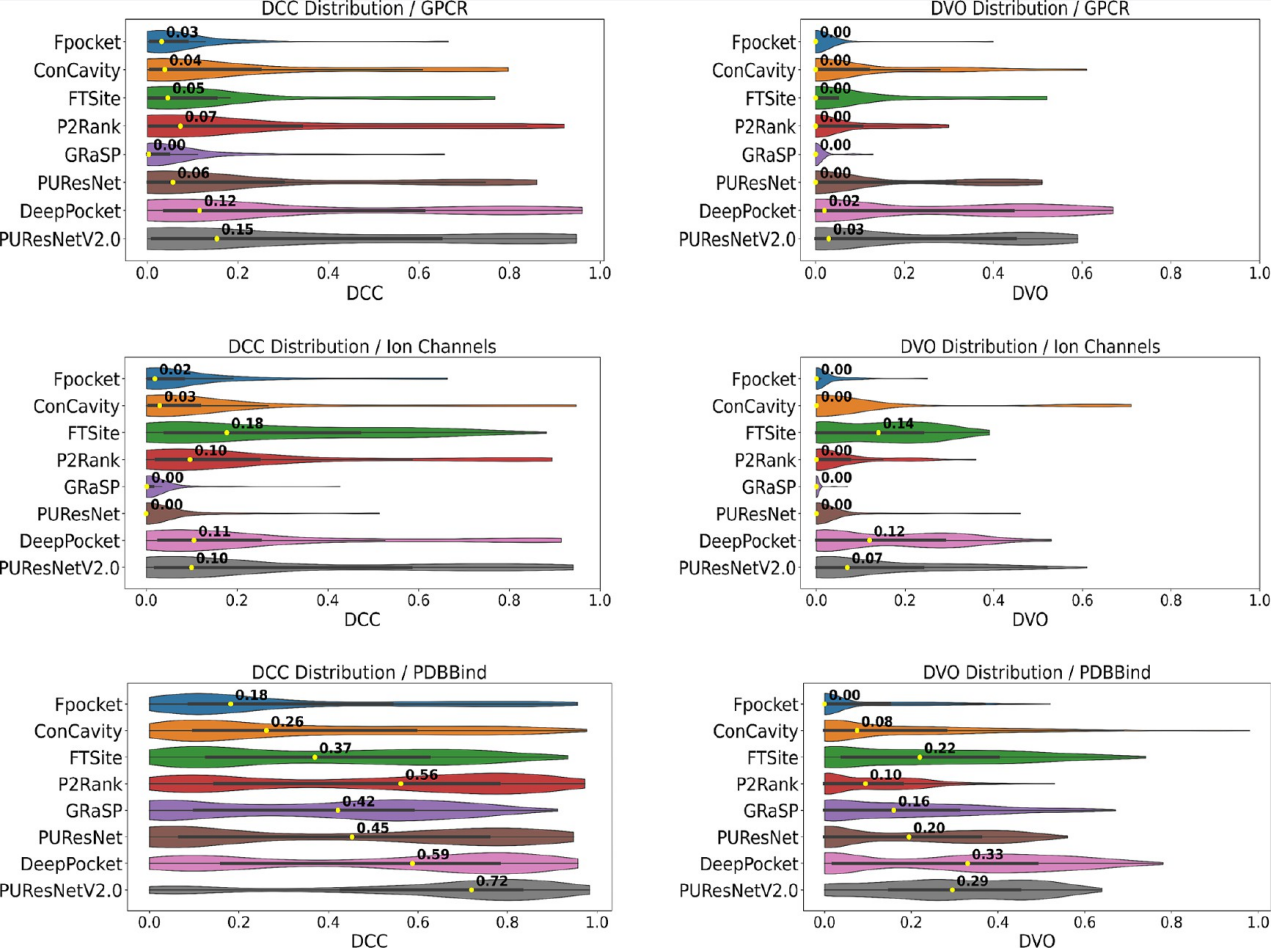
Distribution of the DCC (normalized) and DVO values obtained by the prediction methods across all data sets. Numbers on violins represent the median value across the top-1 predictions.

Looking at the median values across data sets, it is apparent that PUResNetV2.0 is the best-performing method in three cases: for the GPCR data set in the DCC and DVO value distributions and for the PDBBind data set according to the DCC value distribution. However, it is less accurate on the ion channel data set based on median normalized DCC (0.10) and DVO metrics (0.07), which are lower compared to the median values of FTSite (0.18 and 0.14 for normalized DCC and DVO, respectively) and DeepPocket (normalized DCC = 0.11, DVO = 0.12). DeepPocket also performs very well and is the best method on the ion channel and PDBBind data sets according to DVO, the second best on the GPCR data set in terms of both normalized DCC and DVO, and the second best after PUResNetV2.0 on the PDBBind data set when looking at the DCC distribution. FTSite performs particularly well on the ion channel data set, where it is the best method in terms of normalized DCC and the second best method by DVO, shortly after DeepPocket.

GRaSP achieved medium accurate predictions on the PDBBind data set (median DVO = 0.16); however, it was much less successful in the prediction of GPCR and ion channel ligand binding sites. The median DVO value obtained for these data sets was 0.0. A similar observation is made when one looks at the DCC plots. GRaSP ranked on the fifth place for PDBBind with a normalized DCC of 0.42, following PUResNetV2.0 (DCC = 0.72), DeepPocket (DCC = 0.59), P2Rank (DCC = 0.56), and PUResNet (DCC = 0.45). However, on the membrane protein data sets, GRaSP achieved the lowest median DCC values of 0.0 for GPCRs and ion channels.

ConCavity and Fpocket showed comparable low results on membrane proteins. The median DCC value when considering the top-1 predictions of ConCavity was 0.04 and 0.03 for GPCR and ion channel data sets, respectively. The median DVO value of 0.0 confirms that ConCavity’s top-1 predictions failed to correctly predict intramembrane binding sites in GPCRs and ion channels. Fpocket was similarly unsuccessful on membrane proteins, achieving normalized DCC values of 0.03 and 0.02 for GPCR and ion channels, respectively, and a median DVO value of 0.00. Although the performance of ConCavity and Fpocket on the PDBBind data set was slightly better (DCC values of 0.26 and 0.18 and DVO values of 0.08 and 0.00), these methods remained the worst performing ones. This indicates that in case of these methods, looking at the top-1 prediction is not sufficient and that one should consider also the top-3 and top-5 predictions as this increases the success rate, as shown in Figure 7.

PUResNet is fairly successful in predicting binding sites in soluble proteins and achieved median DVO and normalized DCC values of 0.20 and 0.45, respectively, with the top-1 prediction. However, PUResNet was not able to correctly detect the intramembrane binding sites of membrane proteins. The median DVO and DCC values were both 0.00 for ion channels and 0.00 and 0.06 for GPCRs. A possible explanation of PUResNet’s inaccurate results on membrane proteins, in particular, ion channels, is its low sensitivity to multimeric protein structures.

Interestingly, FTSite proved its applicability to predict intramembrane binding sites in ion channels. In contrast to its medium success rates in Figure 7, it achieved the highest median DCC (0.18) and the second highest DVO value (0.14) among the different methods on this data set. However, FTSite failed to correctly predict membrane binding sites in GPCRs (median DVO and DCC of 0.00 and 0.05, respectively), showing again that looking at the top-1 prediction is not sufficient.

P2Rank accomplished negligible DVO and DCC values across membrane protein data sets (DVO of 0.00 for both GPCRs and ion channels; DCC of 0.07 for GPCRs and 0.10 for ion channels), making it less suitable for this prediction task. Not surprisingly, the performance for the PDBBind data set was again better, achieving a median DVO of 0.10 and the third best DCC value of 0.56.

In summary, we can conclude that performance of the currently available binding site prediction methods still lacks the ability to correctly predict pockets in the intramembrane region and that prediction accuracy is considerably worse than for soluble proteins. PUResNetV2.0, DeepPocket, and FTSite are the overall best-performing methods and could serve as starting points for further method improvements by, e.g., fine-tuning them on the constantly increasing number of membrane protein structures.

### Successfully Predicted Intramembrane Binding Sites Tend to Have Higher Binding Site Curvature Values and Less Exposure to the Membrane

In this section, we present examples of successful and unsuccessful binding site predictions of selected GPCR and ion channel structures to indicate possible reasons that may have impacted the prediction accuracy in each case.

In Figure 9, the successful prediction of the intramembrane interface binding site of the antagonist ONO-2570366 in the cysteinyl leukotriene receptor 2 (CysLT_2_R) (PDB: 6RZ7)^102^ is illustrated. The ONO-2570366 ligand binds in the TM3456_ext pocket (according to the nomenclature by Peter et al.^93^), i.e., in the upper part of helices 3, 4, 5, and 6. The upper half of the ligand protrudes between helices 4 and 5 into the intrahelical binding pocket, and the lower molecule half, containing the Cl,F-substituted phenyl ring, is bound on the outer surface of the receptor where it can interact with membrane lipids. It can be seen that all methods were able to correctly identify the binding site position. The reason for the high performance in this case could be due to the fact that the ligand binding site is close to the well-known orthosteric pocket of GPCRs, with half of the molecule being clamped between helices 4 and 5 and the other half being membrane-exposed. Another reason can be the distinct concave shape of the binding pocket.

**Figure 9:**
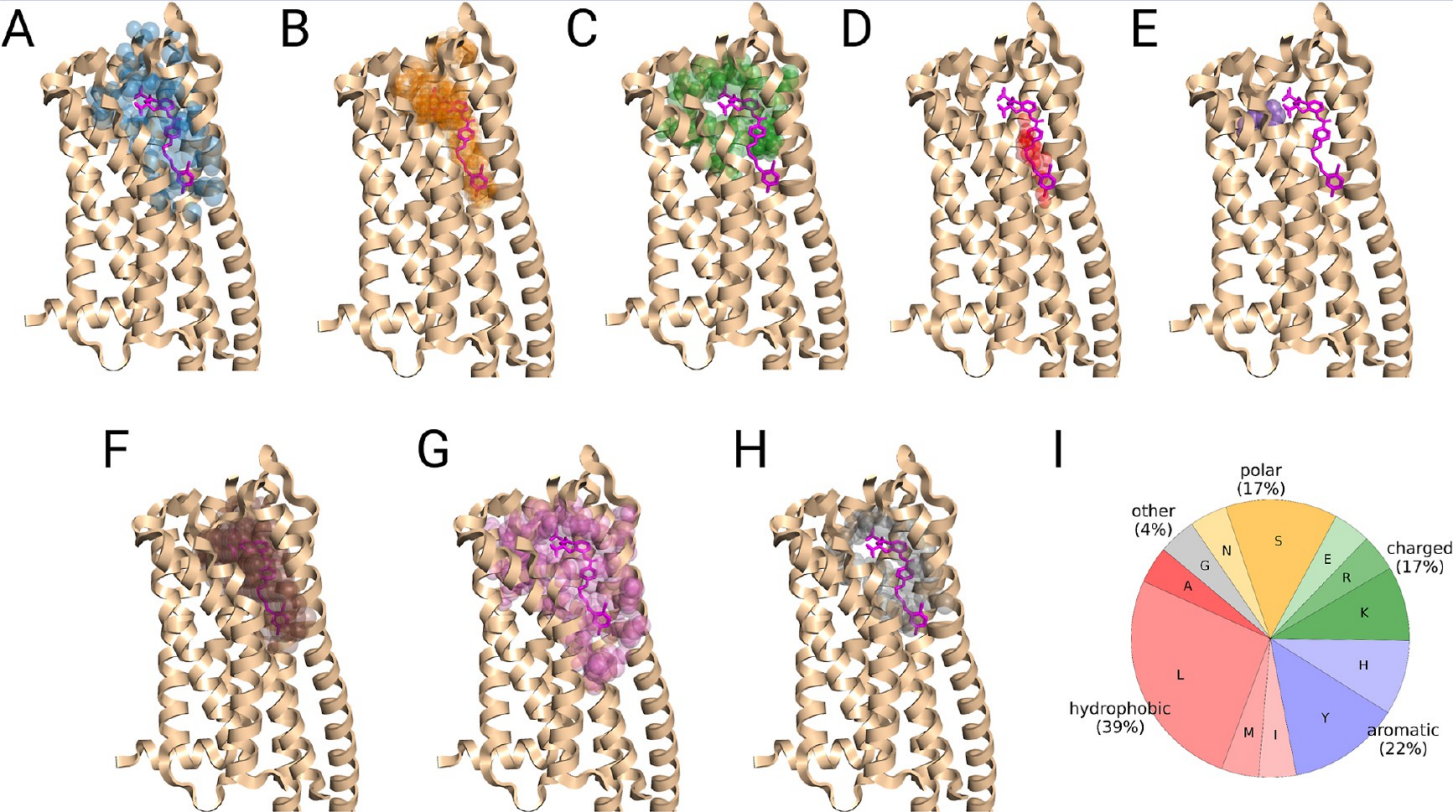
Example of successful binding site predictions (top-1 prediction) made for cysteinyl leukotriene receptor 2 (CysLT_2_R) (PDB: 6RZ7).^102^ The prediction outputs of (A) Fpocket, (B) ConCavity, (C) FTSite, (D) P2Rank, (E) GRaSP, (F) PUResNet, (G) DeepPocket, and (H) PUResNetV2.0 are shown side-by-side. (I) Average amino acid type composition of the ligand binding pocket for CysLT_2_R. The cocrystallized ligand, whose binding site should be predicted, is colored in magenta. Method predictions are shown in colored semitransparent spheres.

In Figure 10, the unsuccessful prediction of the intramembrane interface site of the allosteric antagonist NDT9513727 in the complement C5a receptor 1 (C5aR1) (PDB: 5O9H)^103^ is shown. The ligand binds at the membrane–protein interface in the middle part of the receptor between TM helices 3, 4, and 5 (pocket TM345_mid).^93^ It can be seen that no method was able to correctly identify the position of this pocket. Most predictions are located in the intrahelical region of the GPCR near the known orthosteric ligand binding site. When looking at all predicted pockets for this target, only the top-5 ranked pocket in the case of ConCavity and the top-3 ranked pocket in the case of DeepPocket were able to correctly capture the binding site of NDT9513727, whereas the remaining methods failed to predict it correctly.

**Figure 10:**
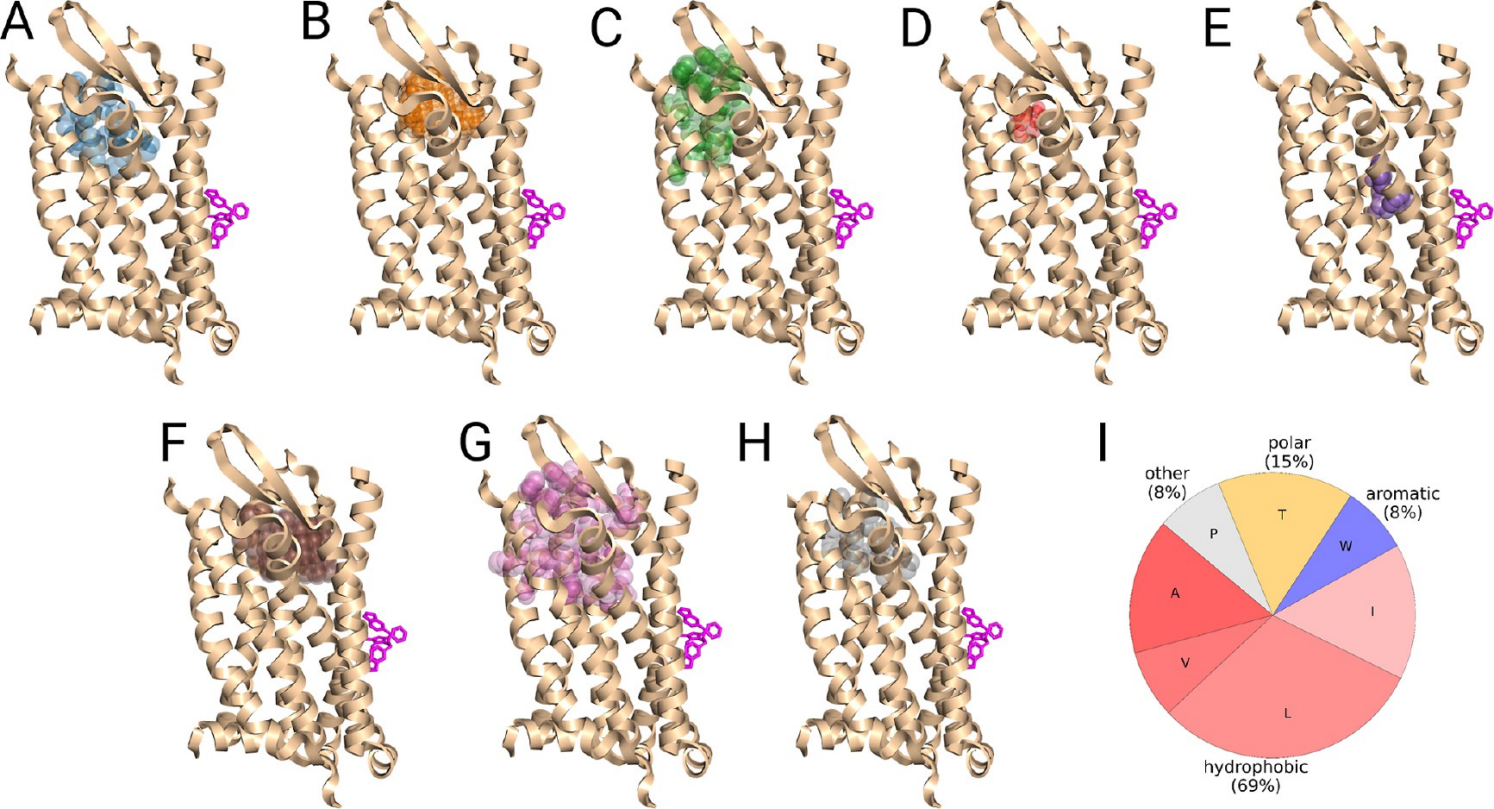
Example of unsuccessful binding site predictions (top-1 predictions) made for complement C5a receptor 1 (PDB: 5O9H).^103^ The prediction outputs of (A) Fpocket, (B) ConCavity, (C) FTSite, (D) P2Rank, (E) GRaSP, (F) PUResNet, (G) DeepPocket, and (H) PUResNetV2.0 are shown side-by-side. (I) Average amino acid type composition of the ligand binding pocket for C5aR1. The cocrystallized ligand, whose binding site should be predicted, is colored in magenta. Method predictions are shown in colored semitransparent spheres.

Another reason for the successful determination of the pocket in the case of CysLT_2_R could be the amino acid composition of the binding site. As we analyzed before, pockets in soluble proteins tend to contain a larger fraction of charged and polar amino acids, whereas hydrophobic and aromatic counterparts are more frequently observed in intramembrane regions. We speculated if successful and unsuccessful predictions differ in residues that compose binding sites. We calculated the average ratios of amino acids present in CysLT_2_R and C5aR1. As illustrated in Figure 9I, CysLT_2_R contains a significant number of charged and polar residues (both 17%), whereas C5aR1 (Figure 10I) does not have any charged residue and has a smaller portion of polar residues (14%). Hydrophobic amino acids constitute a much larger part of the protein binding site in C5aR1 (69%) and only 39% in CysLT_2_R.

Additionally, we noticed that the NDT9513727 molecule binds at the dimeric interface of two C5aR1 molecules in the X-ray structure. Although it was shown that binding of NDT9513727 does not depend on C5aR1 dimerization and can bind to one C5aR1 molecule, the particular binding site observed in the X-ray structure may be formed only in the dimeric complex.^103^ These observations highlight difficulties in the identification of allosteric ligand binding sites. Also the above-mentioned P2Y_1_R ligand BPTU represented a challenging test case, with no method correctly predicting its binding site among the top-7 ranked pockets.

In Figure 11, a successfully predicted case for the transient receptor potential vanilloid member 1 (TRPV1) channel (PDB: 7LPD)^104^ is presented. As can be seen, every method, with the exception of ConCavity, was able to predict the location of the binding site of the JNJ 55511118 antagonist. ConCavity, in contrast, focused its prediction on the channel pore region. The TRPV1 channel ligand is located partially intrahelically, with half of the molecule looking toward the membrane interface. The high degree of pocket curvature could render this binding site more detectable and could have led to a higher probability of success.

**Figure 11:**
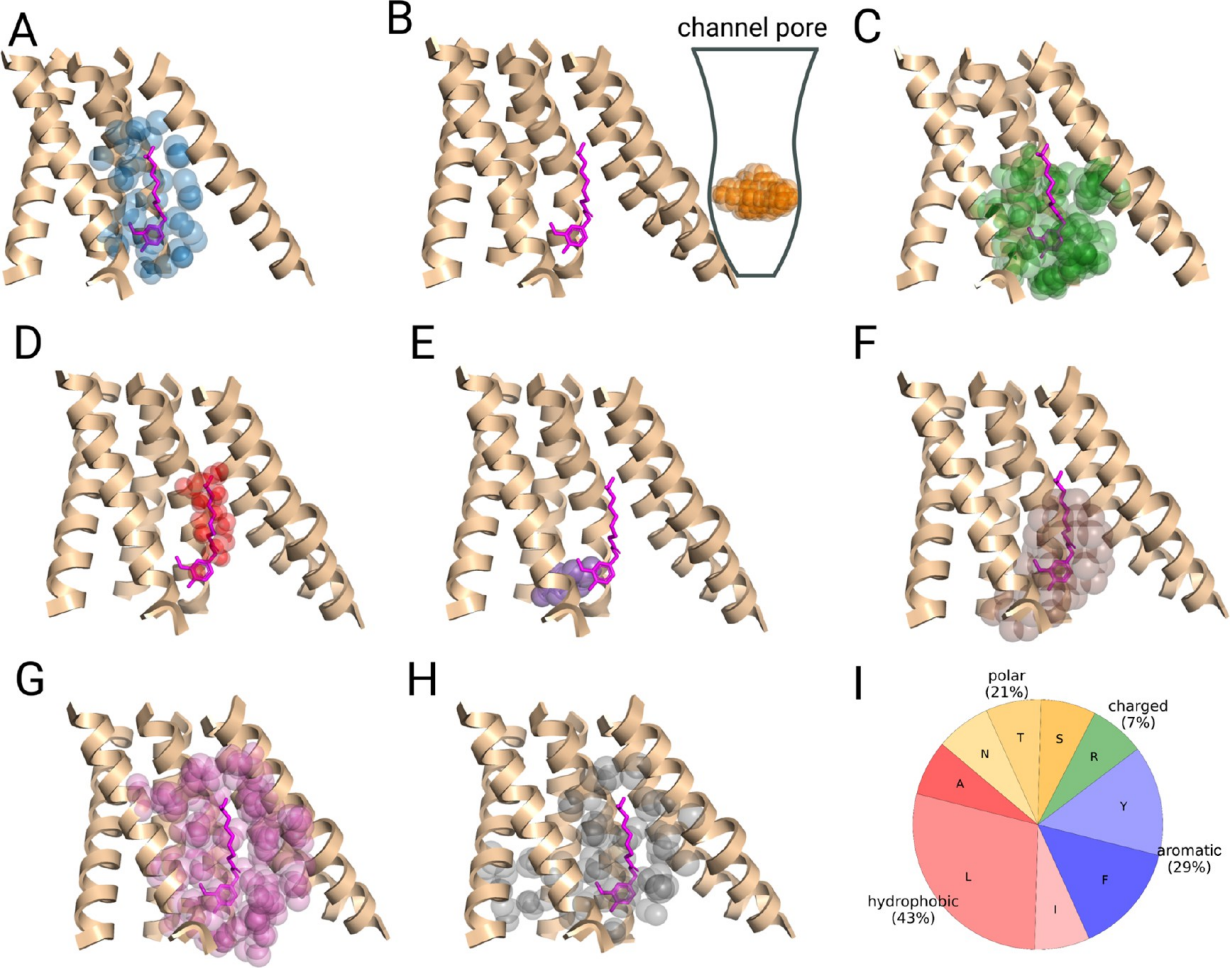
Example of successful binding site predictions (top-1 prediction) made for TRPV1 channel (PDB: 7LPD).^104^ The prediction outputs of (A) Fpocket, (B) ConCavity with a schematic pore region to the right, (C) FTSite, (D) P2Rank, (E) GRaSP, (F) PUResNet, (G) DeepPocket, and (H) PUResNetV2.0 are shown side-by-side. (I) Average amino acid type composition of the ligand binding pocket for TRPV1. The cocrystallized ligand, whose binding site should be predicted, is colored in magenta. Method predictions are shown in colored semitransparent spheres. Only part of the protein in the vicinity of binding site is shown.

According to Figure 12, all methods struggle to predict an intramembrane binding site of the transient receptor potential cation channel subfamily V, member 6 (TRPV6) (PDB: 7S8C).^105^ PUResNet was not able to detect any pockets, and thus, it is not shown in Figure 12. Inhibitor econazole binds between helices S1/S4 of one subunit and S5 of the neighboring subunit, overlapping with a natural lipid binding site of the channel. Fpocket, ConCavity, and FTSite tend to predict the central pore as the most probable binding site, which is a known pocket for ion channel pore blockers (e.g., ruthenium red).^105^ All other methods detected pockets in the intrahelical region within one subunit, whereas the binding site of econazole is located at the protein–membrane interface between two neighboring subunits and was overlooked by all methods. The binding site, which was predicted, is known to be bound by 2-aminoethoxydiphenyl borate and has been previously well characterized.^106^ It is worth noting that several methods (e.g., DeepPocket) identified the binding site of econazole, albeit not among top predictions.

**Figure 12:**
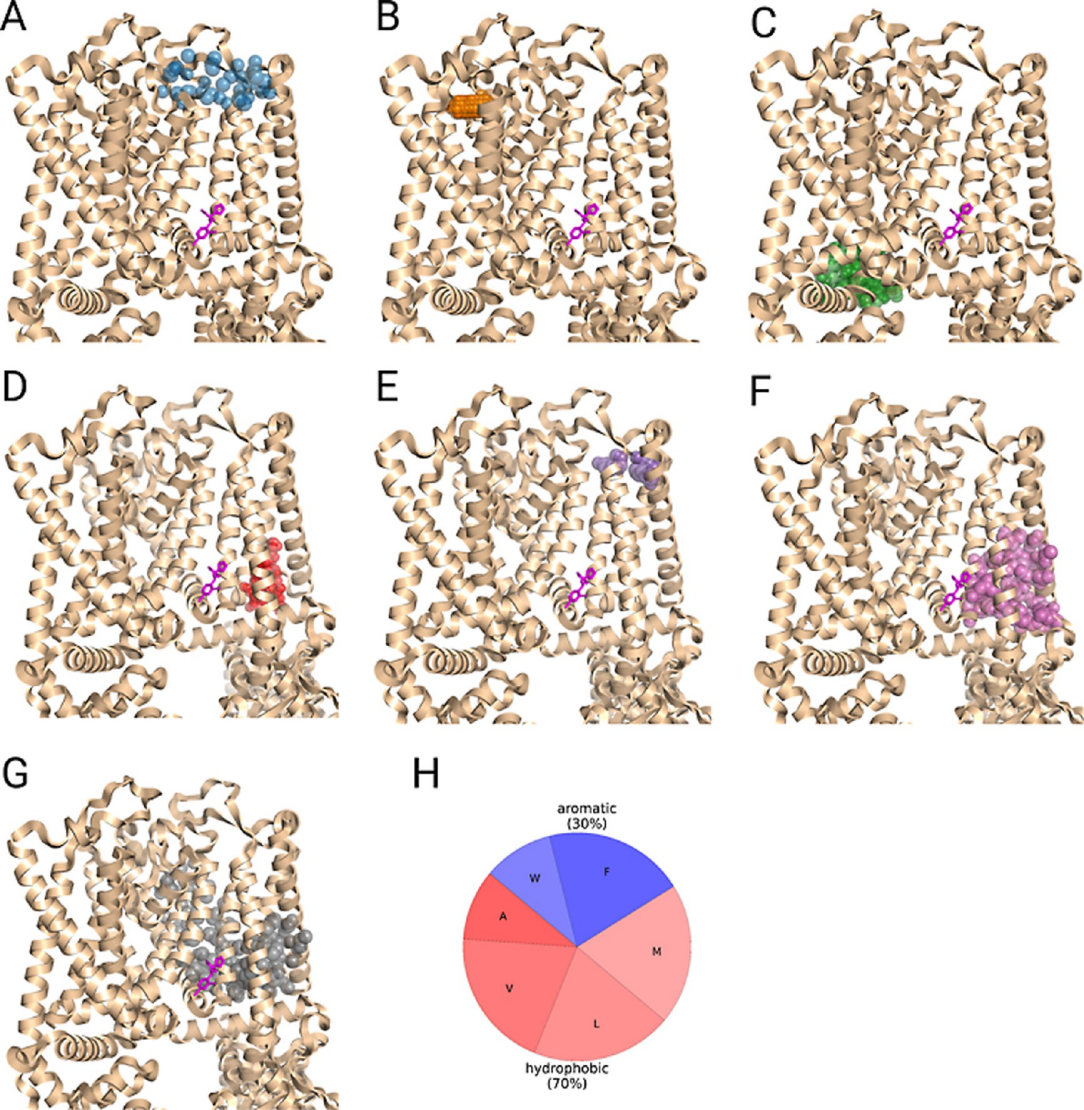
Example of unsuccessful binding site predictions (top-1 predictions) made for transient receptor potential cation channel subfamily V, member 6 (TRPV6) (PDB: 7S8C).^105^ The prediction outputs of (A) Fpocket, (B) ConCavity, (C) FTSite, (D) P2Rank, (E) GraSP, (F) DeepPocket, and (G) PUResNetV2.0 are shown side-by-side. (I) Average amino acid type composition of the ligand binding pocket for TRPV6. The cocrystallized ligand, whose binding site should be predicted, is colored in magenta. Method predictions are shown in colored semitransparent spheres. Only part of the transmembrane region of the protein is shown.

As for the comparison of CysLT_2_R and C5aR1 above, we additionally calculated the amino acid composition of TRPV1 and TRPV6 ligand binding pockets to evaluate reasons for their different success rates. As shown in Figure 12I, the pocket of TRPV6 exclusively consists of hydrophobic and aromatic residues (70% and 30%, respectively), whereas TRPV1 has a significant fraction of polar (21%) and some charged (7%) residues within its intramembrane binding site (Figure 11I). It should not necessarily be the primary reason for the success rates seen in this study; nevertheless, the differences in amino acid composition are observed throughout all three data sets.

Additionally, we performed a pocket comparison of the GPCR and ion channels in terms of their binding site areas. We conducted a simple calculation in PyMOL,^107^ with its get_area function, which calculates the surface area of a selected protein region.^108^ Binding pockets span a wide size range, from 50 Å^2^ up to 1200 Å^2^ in GPCRs and 100 Å^2^ up to 1000 Å^2^ in ion channels, with a median size of 600 Å^2^ (Supporting Information Figures S2 and S3). However, no difference in binding pocket sizes between successful and unsuccessful prediction cases could be found.

Another property that could affect success rates is the surface curvature. To investigate the relationship between successful and unsuccessful cases, we computed the curvature values of binding sites across all data sets with the Surface Racer software.^109^ To obtain the curvature of the binding sites, we calculated the average curvature of residues that build up the binding site. Negative curvature values indicate a convex surface (i.e., outward-curving), whereas positive values show a concave binding site (i.e., inward-curving). As can be seen in Supporting Information Figures S4–S6, all methods exhibit higher number of successful cases on concave binding sites. Conversely, as the curvature becomes more convex, there is a decline in method performance. Furthermore, we observed that proteins in the PDBBind data set exhibit a higher number of concave binding sites in comparison to those in membrane proteins. This in turn could be one reason for the higher success rate on soluble proteins.

Looking at the four prediction cases mentioned above, CysLT_2_R was found to have a binding site with a size of 866 Å^2^ and a high inward curvature of 0.157 Å^–1^. In comparison, C5aR1 exhibits a slightly lower binding site area of 695 Å^2^ and a less inward curvature of 0.069 Å^–1^, which could explain the lower method performance for C5aR1. TRPV1 has a binding site area size of 400 Å^2^ and a high inward curvature of 0.160 Å^–1^. Although the area and the surface curvature of the TRPV6 binding site were found to be similar (451 Å^2^ and 0.15 Å^–1^, respectively), there is a slight decline in the curvature value. An important influence of binding site curvature on the success rate of various prediction methods is expected, particularly for geometry-based methods. This finding highlights the importance of accounting for differences in binding site geometry when evaluating the effectiveness of computational methods in predicting intramembrane binding sites.

Another factor that can influence prediction performance is the receptor’s activation state. Previously, Peter at al.^93^ observed higher success rates for GPCRs when the structure was in an inactive conformation. To further investigate this effect, we divided our GPCR data set according to the structure’s activation state and calculated success rates separately for active-state (21 proteins) and inactive-state structures (18 proteins). Our results are consistent with those reported in Peter at al.,^93^ showing higher success rates in the group of inactive-state structures compared to their active counterparts (Supporting Information Figure S7). Albeit being an interesting observation that requires further investigation, it is not advisable to use exclusively GPCR structures in inactive states to improve success chances but to still consider the biologically most relevant receptor state.

## Conclusion

This study represents the first comprehensive benchmarking of computational binding site prediction methods on transmembrane proteins, focusing specifically on the prediction of binding sites at the protein–membrane interface. A growing body of research shows that GPCRs and ion channels can bind small-molecule ligands at unexpected sites on their exterior membrane-exposed surface, which can have important activity-modulating functions. Oftentimes these sites have been discovered by chance, and there are probably more sites that still await to be discovered. Harnessing these sites in GPCR or ion channel drug discovery should be highly valuable because of their modulatory functions, but it requires systematic and reliable approaches for detecting these sites.

Our results show that all computational methods perform relatively poorly on this task and have significantly lower success rates for membrane proteins than for soluble proteins. The methods with the average best performance are PUResNetV2.0, DeepPocket, and FTSite. These methods can achieve acceptable results, although one should carefully analyze their predictions, bearing in mind that they were not developed specifically for membrane proteins.

To advance basic and applied research on GPCRs and ion channels, future developments can focus on optimizing these methods for membrane proteins. Possible directions could include the use of transfer learning by introducing membrane-specific layers into current deep learning architectures, which could enhance the accuracy for membrane-exposed binding sites.^110–112^ Another possibility could be the design of specific molecular interaction probes, which would be tailored to the chemical composition of membrane-exposed binding sites and could improve the performance of energy-based binding site prediction methods.^113,114^ Furthermore, the relative affinity of a drug to bilayer lipids, compared to that of the aqueous phase, influences its accessibility and transfer to the protein–membrane interface. Thus, membrane partitioning information on small molecules can be utilized in machine learning methods to enhance binding site prediction.^115^

## Supplementary Material



## Data Availability

The data sets of GPCR and ion channel structures created in this study, the subset of 420 PDBBind structures as well as Python scripts used to download PDB files from OPM database and clean them from all unwanted ligands, and to handle predictions can be obtained from Zenodo at https://doi.org/10.5281/zenodo.14918827 and from GitHub at https://github.com/kuenzelab/binding_sites_prediction.

## Notes

Tables of GPCR, ion channel, and PDBBind structures used in this study; overview of binding site prediction methods found in scientific literature; examples of ligands used to represent GPCR binding sites; top-7 DCC and DVO success rates of ligand binding site prediction methods across all data sets; bar plots of performance comparison of binding site prediction methods across used data sets in terms of top-1 and top-7 success rates; bar plots showing distribution of successful and unsuccessful cases per binding site area range in GPCR, ion channel, and PDBBind data sets; bar plots showing distribution of successful and unsuccessful cases per binding site surface curvature in GPCR, ion channel, and PDBBind data sets; and bar plots showing performance comparison of binding site prediction methods tested on active and inactive conformations of GPCRs (PDF)
