# Supplementary material for: Evaluation of Small-Molecule Binding Site Prediction Methods on Membrane-Embedded Protein Interfaces
Source: J Chem Inf Model. 2025 Jul 2;65(13):6949–67. doi: 10.1021/acs.jcim.5c00336 (PMC12264969; doi:10.1021/acs.jcim.5c00336)
Supplement: Supplementary file 1 [file ci5c00336_si_001.pdf]

Supporting Material for

Evaluation of small-molecule binding site prediction methods on membrane-embedded protein interfaces

Palina Pliushcheuskaya[1], *Georg Künze*[1,2,3,*]

[1] Institute for Drug Discovery, Medical Faculty, University of Leipzig, Leipzig 04103, Germany

[2] Interdisciplinary Center for Bioinformatics, University of Leipzig, Leipzig 04107, Germany

[3] Center for Scalable Data Analytics and Artificial Intelligence, University of Leipzig, Leipzig 04105, Germany

[*] Author to whom correspondence should be addressed: georg.kuenze@uni-leipzig.de

SUPPORTING METHODS

Binding site surface curvature calculation

The Surface Racer software was installed on a local OS X computer utilizing its source web page.[2] The program is run by calling its executable file from the command line. Surface Racer accepts three user interactive inputs upon running:

I. Analytical surface area calculation: One can choose between the van der Waals radii sets of Richards (1977) or Chothia (1976).

II. Input PDB file of the structure: query pdb file.

III. Input the probe radius in Angstroms: Can be either 1.2 Å or 1.4 Å.

In our calculations we used the van der Waals radii set from Richards[3] to describe atoms (option I), and a probe with radius of 1.4 Å (option III), which rolls over the protein surface to calculate the accessible surface area, which is then used to compute curvature values for individual atoms and amino acid residues.

SUPPORTING TABLES

Table S1. GPCR structures used in the study. TM – transmembrane helix, ext – exterior, int – interior, mid – middle sites.

PDB Code	Protein Name	Ligand Name	Ligand Function	Pocket	Pocket Location
4XNV	P2Y purinoceptor 1	BPTU	Allosteric antagonist	TM123_ext	Upper part of 1,2,3
7XZ6	Cannabinoid receptor-like GPR119	APD668	Agonist	TM1234567_ext	Upper part of helices 1,2,3,4,5,6,7
6X19	Glucagon-like peptide-1 receptor	CHU-128	Agonist	TM123567_ext	Upper part of helices 1,2,3,5,6,7
7S8P	MAS-related G protein-coupled receptor X (MRGPRX)	MS47134	Agonist	TM123567_ext	Upper part of helices 1,2,3,5,6,7
7WCM	Cannabinoid receptor-like GPR119	MBX-2982	Agonist	TM123567_ext	Upper part of helices 1,2,3,5,6,7
5TGZ	Cannabinoid Receptor CB1	AM6538	Antagonist	TM12367_ext	Upper part of helices 1,2,3,6,7
6IIU	Thromboxane A2 receptor	Ramatroban	Antagonist	TM12367_ext	Upper part of helices 1,2,3,6,7
7YXA	Sphingosine 1 phosphate receptor 5	ONO-5430608	Inverse agonist	TM12367_ext	Upper part of helices 1,2,3,6,7
6GPX	CC chemokine receptor 2	MK-0812	Antagonist	TM1237_ext	Upper part of helices 1,2,3,7
7CX3	Prostaglandin E 2 (PGE 2) receptor EP2	Taprenepag	Agonist	TM1237_ext	Upper part of helices 1,2,3,7
7LCJ	Glucagon-like peptide-1 receptor	PF 06882961	Agonist	TM1237_ext	Upper part of helices 1,2,3,7
7EPE	Metabotropic glutamate receptor 2	CHEMBL3894759	Negative allosteric modulator	TM234567_ext	Upper part of helices 2,3,4,5,6,7
7SIL	Extracellular calcium-sensing (CaS) receptor	3-(2-chlorophenyl)-N-[(1R)-1-(3-methoxyphenyl)ethyl]propan-1-amine	Positive allosteric modulator	TM234567_ext	Upper part of helices 2,3,4,5,6,7
5TZR	Free fatty acid receptor 1	MK-8666	Partial agonist	TM3456_ext	Upper part of helices 3,4,5,6
6FK6	Rhodopsin	(2~{S})-2-(4-chlorophenyl)-3-methyl-1-spiro[1,3-benzodioxole-2,4'-piperidine]-1'-yl-butan-1-one	Agonist	TM3456_ext	Upper part of helices 3,4,5,6

6RZ5	Cysteinyl leukotriene receptor 1	Zafirlukast	Antagonist	TM3456_ext	Upper part of helices 3,4,5,6
6RZ7	Cysteinyl leukotriene receptor 2	ONO-2570366	Antagonist	TM3456_ext	Upper part of helices 3,4,5,6
8EJC	Free fatty acid receptor 1	Fasiglifam	Agonist	TM3456_ext	Upper part of helices 3,4,5,6
7M3G	Extracellular calcium-sensing receptor	Evocalcet	Postive allosteric modulator	TM4567_ext	Upper part of helices 4,5,6,7
7UL3	Histamine receptor 2	Famotidine	Antagonist	TM4567_ext	Upper part of helices 4,5,6,7
8J6P	Hydroxycarboxylic acid receptor 2	MK-6892	Positive allosteric modulator	TM56_ext	Upper part of helices 5,6
7P2L	Metabotropic glutamate receptor (mGlu5)	2-chloranyl-~{N}-[2-methoxy-4-[(~{E})-pyridin-2-yldiazenyl]phenyl]benzamide	Negative allosteric modulator	TM567_ext	Upper part of helices 5,6,7
4K5Y	Corticotropin-releasing factor receptor 1	CP-376395	Antagonist	TM23567_mid	Middle part of helices 2,3,5,6,7
7FEE	Cannabinoid receptor CB1	ZCZ011	Positive allosteric modulator	TM34_mid	Middle part of helices 3,4
5KW2	Free fatty acid receptor 1	(3~{S})-3-cyclopropyl-3-[2-[1-[2-[2,2-dimethylpropyl-(6-methylpyridin-2-yl)carbamoyl]-5-methoxy-phenyl]piperidin-4-yl]-1-benzofuran-6-yl]propanoic acid	Allosteric agonist	TM345_mid	Middle part of helices 3,4,5
5O9H	Complement component 5a receptor 1	NDT9513727	Antagonist	TM345_mid	Middle part of helices 3,4,5
6C1Q	C5a receptor	NDT9513727	Negative allosteric modulator	TM345_mid	Middle part of helices 3,4,5
7C7Q	GABA(B) receptor	(3S)-5,7-ditert-butyl-3-oxidanyl-3-(trifluoromethyl)-1-benzofuran-2-one	Positive allosteric modulator	TM456_mid	Middle part of helices 4,5,6
5T1A	CC chemokine receptor 2	CCR2-RA-[R]	Allosteric antagonist	TM124678_int	Lower part of helices 1,2,4,6,7,8
5X7D	β2 adrenergic receptor	4-carbamoyl-N-[(2R)-2-cyclohexyl-2-phenylacetyl]-L-phenylalanyl-3-bromo-N-methyl-L-phenylalaninamide	Negative allosteric modulator	TM23678_int	Lower part of helices 2,3,6,7,8
6QZH	C-C chemokine receptor type 7	Cmp2105	Allosteric antagonist	TM23678_int	Lower part of helices 2,3,6,7,8
7EJX	GPR88-Gi1	(1R,2R)-N-[(2S,3S)-2-azanyl-3-methyl-pentyl]-N-[4-(4-propylphenyl)phenyl]-2-pyridin-2-yl-cyclopropane-1-carboxamide	Allosteric agonist	TM23678_int	Lower part of helices 2,3,6,7,8

5EE7	Glucagon receptor	MK-0893	Allosteric antagonist	TM2678_int	Lower part of helices 2,6,7,8
5VEW	Glucagon-like peptide-1 receptor	PF-06372222	Negative allosteric modulator	TM2678_int	Lower part of helices 2,6,7,8
5XEZ	Glucagon receptor	NNC0640	Negative allosteric modulator	TM2678_int	Lower part of helices 2,6,7,8
6KJV	Glucagon-like peptide-1 receptor	N-{4-[(R)-(3,3-dimethylcyclobutyl)({6-[4-(trifluoromethyl)-1H-imidazol-1-yl]pyridin-3-yl}amino)methyl]benzene-1-carbonyl}-beta-alanine	Negative allosteric modulator	TM2678_int	Lower part of helices 2,6,7,8
7LJC	D1 dopamine receptor	Mevidalen	Positive allosteric modulator	TM34_int	Lower part of helices 3,4
7DUQ	Glucagon-like peptide-1 receptor	N-tert-butyl-6,7-bis(chloranyl)quinoxalin-2-amine	Ago-allosteric modulator	TM6_int	Lower part of helix 6
7EVM	Glucagon-like peptide-1 receptor	N-Tert-butyl-6,7-dichloroquinoxalin-2-amine	Positive allosteric modulator	TM6_int	Lower part of helix 6

Table S2. Ion channels structures used in the study.

PDB Code	Protein Name	Ligand Name	Ligand Function
4NTX	Acid-sensing ion channel	Amiloride	Inhibitor
4XDL	Potassium Two Pore Domain Channel Subfamily K Member 10 (KCNK19)	Fluoxetine (Prozac)	Inhibitor
5DQQ	Two-pore channel TPC1	trans-NED19	Inhibitor
5EK0	Voltage-gated sodium channel ($Na_V1.7$)	GX-936	Inhibitor
5IS0	Transient receptor potential cation channel, subfamily V, member 1 (TRPV1)	Capsazepine	Antagonist
5KLS	Voltage-gated calcium channel (Ca_VAb)	UK-59811	Antagonist
6B5V	Transient receptor potential cation channel subfamily V, member 5 (TRPV5)	Econazole	Inhibitor
6B21	Ammonia channel (AmtB)	TopFluor cardiolipin	Positive allosteric modulator
6CQ8	Two-pore domain potassium channel, TREK subfamily ($K_{2P}2.1$)	ML335	Activator

6DVY	Transient receptor potential cation channel, subfamily V, member 3 (TRPV3)	2-Aminoethoxydiphenyl borate (2-APB)	Activator
6JP5	Voltage-dependent L-type calcium channel (Ca_V1.1)	Nifedipine	Antagonist
6KZP	Voltage-dependent T-type calcium channel (Ca_V3.1)	Z944	Antagonist
6NR3	Transient receptor potential melastatin member 8 (TRPM8)	Icilin	Activator
6O6R	Transient receptor potential melastatin member 8 (TRPM8)	N-(3-aminopropyl)-2-[(3-methylphenyl) methoxy] -N-(2-thienylmethyl) benzamide hydrochloride (AMTB)	Antagonist
6O9T	Inward rectifier K^+ channel (Kir)	2,3,5,6-tetramethyl-1H,7H-pyrazolo[1,2-a]pyrazole-1,7-dione	Activator
6O72	Transient receptor potential melastatin member 8 (TRPM8)	TC-I 2014	Antagonist
6OO4	Transient receptor potential cation channel subfamily V member 2 (TRPV2)	Resiniferatoxin (RTx)	Agonist
6PBE	Transient receptor potential cation channel subfamily V member 5 (TRPV5)	ZINC17988990	Inhibitor
6RUQ	AMPA receptor	GYKI53655	Negative allosteric modulator
6U88	Transient receptor potential cation channel subfamily V member 2 (TRPV2)	Cannabidiol	Activator
6UIW	Calcium homeostasis modulator	Ruthenium red (RUR)	Inhibitor
6UZ8	Transient receptor potential cation channel, subfamily C, member 6 (TRPC6)	AM-0883	Agonist
6UZA	Transient receptor potential cation channel, subfamily C, member 6 (TRPC6)	AM-1473	Antagonist
6VQR	*Plasmodium falciparum* formate–nitrite transporter (PfFNT)	MMV007839	Inhibitor
6WKN	Transient receptor potential cation channel subfamily V member 2 (TRPV2)	Iperlongumine	Allosteric antagonist
6X3X	$GABA_A$ receptor	Diazepam	Positive allosteric modulator
6XSR	AMPA receptor	trans-4-butylcyclohexane carboxylic acid (4-BCCA)	Inhibitor
7B05	Transient receptor potential cation channel, subfamily C, member 4 (TRPC4)	GFB-8749	Inhibitor

7BYM	Voltage-gated potassium channel KCNQ4	Retigabine	Activator
7CR1	Voltage-gated potassium channel KCNQ2	ztz240	Activator
7CR7	Voltage-gated potassium channel KCNQ2	Retigabine	Activator
7D2K	Transient receptor potential cation channel, subfamily V, member 6 (TRPV6)	Br-cis-22a	Inhibitor
7D4P	Transient receptor potential cation channel, subfamily C, member 5 (TRPC5)	Clemizole	Inhibitor
7D4Q	Transient receptor potential cation channel, subfamily C, member 5 (TRPC5)	HC-070	Inhibitor
7DXF	Transient receptor potential cation channel, subfamily C, member 6 (TRPC6)	BTDM	Inhibitor
7DXG	Transient receptor potential cation channel, subfamily C, member 6 (TRPC6)	SAR7334	Inhibitor
7JPK	Voltage-gated calcium channel ($Ca_V1.1$)	(S)-(-)-Bay K8644	Allosteric modulator
7K4B	Transient receptor potential cation channel, subfamily V, member 6 (TRPV6)	cis-22a	Inhibitor
7LDD	AMPA receptor	JNJ 55511118	Antagonist
7LID	Olfactory receptor	Eugenol	Agonist
7LPD	Transient receptor potential cation channel, subfamily V, member 1 (TRPV1)	Capsaicin	Activator
7LQZ	Transient receptor potential cation channel, subfamily V, member 1 (TRPV1)	Resiniferatoxin (RTx)	Agonist
7MGL	Transient receptor potential mucolipin 1 (TRPML1)	ML-SI3	Inhibitor
7RAU	Transient receptor potential cation channel, subfamily V, member 3 (TRPV3)	Osthole	Inhibitor
7S8C	Transient receptor potential cation channel subfamily V, member 6 (TRPV6)	Econazole	Inhibitor
7TCI	Voltage-gated potassium channel KCNQ1 ($K_V7.1$)	ML277	Activator
7U1Q	ATP-sensitive potassium channel (K_{ATP})	Repaglinide	Inhibitor
7UGG	Transient receptor potential cation channel, subfamily V, member 3 (TRPV3)	Dyclonine	Inhibitor
7VNQ	Voltage-gated potassium channel KCNQ4	ML213	Activator

7W4O	ATP-sensitive potassium channel (K_{ATP})	6-chloranyl-~{N}-(1-methylcyclopropyl)-1,1-bis(oxidanylidene)-4~{H}-thieno[3,2-e][1,2,4]thiadiazin-3-amine	Activator
7W7F	Voltage-gated sodium channel $Na_V1.3$	ICA121431	Antagonist
7WDB	Transient receptor potential cation channel, subfamily C, member 5 (TRPC5)	Riluzole	Activator
7XEV	Transient receptor potential cation channel, subfamily V, member 2 (TRPV2)	2-aminoethyl diphenylborinate (2-APB)	Activator
7XJ0	Transient receptor potential cation channel, subfamily V, member 3 (TRPV3)	Trpvicin	Antagonist
8E4M	Transient receptor potential melastatin 8 channel (TRPM8)	Cryosim-3	Agonist
8QUC	Voltage-gated potassium channel K_V3	(5R)-5-ethyl-3-[6-(3-methoxy-4-methyl-phenoxy)pyridin-3-yl]imidazolidine-2,4-dione (AUT1)	Positive allosteric modulator
8SP8	Transient receptor potential cation channel, subfamily V, member 6 (TRPV6)	Tetrahydrocannabivarin	Inhibitor
9B6I	Transient receptor potential melastatin 8 channel (TRPM8)	TC14	Antagonist
9IK1	P2X purinoceptor 3 (P2X3R)	Compound 26a	Antagonist

Table S3. PDB IDs of structures from PDBBind subset.

5qj2, 5y7k, 5yy6, 5z1e, 5z66, 5zbz, 5zcu, 5zdc, 5zdp, 5ze6, 5zee, 5zg3, 5zgg, 5zjf, 5zk7, 5zlf, 5znc, 5zqo, 5zqu, 5zr3, 5zyl, 6a04, 6a3n, 6a6k, 6a73, 6a80, 6a93, 6a9o, 6aa4, 6abk, 6abx, 6agt, 6ahs, 6ai9, 6ajr, 6akw, 6bqd, 6byz, 6c3l, 6c5j, 6c85, 6c8c, 6c9p, 6c9r, 6cf7, 6cjs, 6ckl, 6cq1, 6d3g, 6d3q, 6d49, 6d4r, 6d5w, 6d8v, 6d9s, 6dd0, 6det, 6dha, 6die, 6dsp, 6dtn, 6duk, 6dyn, 6dyy, 6dz3, 6e13, 6e3g, 6e3s, 6e3z, 6e4v, 6e5b, 6e5s, 6e6j, 6e7s, 6edl, 6f7i, 6f86, 6fe5, 6fgc, 6fh3, 6fmn, 6fne, 6ftf, 6fym, 6fzf, 6fzm, 6g2b, 6g2s, 6g3c, 6g5u, 6g7a, 6g9f, 6g9x, 6gbw, 6gcl, 6gcw, 6gdy, 6gfm, 6ggb, 6ghv, 6gj8, 6gjb, 6gjw, 6gvh, 6gvx, 6gwe, 6gxg, 6gxq, 6gzy, 6h0u, 6h1d, 6h4d, 6h4q, 6h4u, 6h4z, 6h75, 6h7d, 6h9v, 6h9x, 6haj, 6hay, 6haz, 6hb5, 6hb7, 6hbm, 6hck, 6hcw, 6hgg, 6hgr, 6hgy, 6hk3, 6hky, 6hm1, 6hmr, 6hmy, 6hni, 6hp0, 6hp9, 6hq3, 6hq7, 6hro, 6hrz, 6htn, 6hv5, 6hx1, 6hy7, 6hys, 6hzc, 6hzv, 6hzy, 6i0r, 6i0x, 6i0z, 6i1r, 6i3u, 6i5g, 6i61, 6i74, 6i8m, 6i96, 6i9a, 6iae, 6iar, 6ibk, 6ibz, 6ift, 6ijh, 6ijl, 6ind, 6inz, 6ior, 6ipi, 6iqg, 6itj, 6iyw, 6izq, 6j0g, 6j0k, 6j10, 6j1l, 6j3o, 6j63, 6j72, 6j7l, 6jam, 6jav, 6jdi, 6jfk, 6jib, 6jio, 6jjm, 6jki, 6jmf, 6jn3, 6jn4, 6jno, 6jon, 6jsg, 6jtc, 6jut, 6jz0, 6k1q, 6k1s, 6k2n, 6k4t, 6kjd, 6klk, 6knh,

6m7h, 6m87, 6m8a, 6m8q, 6m95, 6maj, 6max, 6mbp, 6md6, 6md7, 6me2, 6mep, 6mh1, 6mhb, 6mhc, 6mhm, 6mjq, 6mju, 6mla, 6mlj, 6mlw, 6mm2, 6mm5, 6mmo, 6mnc, 6mo0, 6mo5, 6mom, 6msa, 6mso, 6mt0, 6mxb, 6mxc, 6mxd, 6myn, 6n0j, 6n0p, 6n3k, 6n3l, 6n3v, 6n3y, 6n4b, 6n4e, 6n54, 6n5c, 6n69, 6n6o, 6n78, 6n7a, 6n87, 6n92, 6n97, 6n9h, 6n9l, 6n9p, 6ncj, 6ncn, 6nd3, 6ndl, 6ne5, 6nel, 6nfn, 6ng0, 6nj0, 6njh, 6nlj, 6nm4, 6nmb, 6nng, 6np2, 6npm, 6nss, 6nt2, 6nu1, 6nvl, 6nwk, 6ny0, 6nyv, 6nze, 6nzk, 6nzm, 6nzp, 6nzt, 6o1g, 6o4w, 6o5t, 6o5x, 6o95, 6o9x, 6oag, 6ob0, 6oco, 6ocq, 6ocz, 6oe3, 6of5, 6oh3, 6oi9, 6oja, 6oko, 6om4, 6om8, 6ooz, 6orr, 6os5, 6ott, 6ouv, 6ow7, 6ox0, 6oyh, 6oyw, 6p1l, 6p3t, 6p5o, 6p83, 6p8x, 6p9e, 6peb, 6peg, 6pf3, 6pfj, 6pg8, 6pht, 6phx, 6pi5, 6pic, 6plg, 6pm9, 6pml, 6ppy, 6prg, 6pve, 6pvu, 6py0, 6pya, 6q30, 6q35, 6q37, 6q3y, 6q4q, 6q54, 6q5b, 6q7v, 6q89, 6q8p, 6q9l, 6q9q, 6q9w, 6qas, 6qav, 6qcj, 6qdx, 6qed, 6qhg, 6qi4, 6qi7, 6qm7, 6qmr, 6qmu, 6qpl, 6qr4, 6qre, 6qri, 6qwi, 6qxd, 6qxs, 6qyn, 6qzh, 6r1a, 6r1b, 6r49, 6r4s, 6r4v, 6r7d, 6r7r, 6r8l, 6r8r, 6rj2, 6rj6, 6rmf, 6rn6, 6rnu, 6rpg, 6rqk, 6rtn, 6rz6, 6s0e, 6s1s, 6s55, 6s7k, 6s9w, 6saf, 6sfc, 6sge, 6skb, 6ssy, 6st3, 6sy7, 6szj, 6szp, 6t1i, 6t6a, 6te6, 6tld, 6u26, 6u8b, 6u9v, 6ueg, 6uf0, 6ufo, 6un1, 6v1c,

Table S4. Binding site prediction methods.

Name	Year	Type	Availability
POCKET[4]	1992	Geometric	Software available upon written request
SURFNET[5]	1995	Geometric	Software available upon written request
LIGSITE[6]	1997	Geometric	Algorithm[7]
PASS[8]	2000	Geometric	Not available
CASTp[9]	2003	Geometric	Web server[10]
Q-SiteFinder[11]	2005	Energetic	Not available
LIGSITEcsc[12]	2006	Geometry + Conservation	Not available
SiteFinder[13,14]	2007	Geometric	MOE software[15]
firestar[16]	2007	Template	Web server (accepts single chain)
PocketPicker[17]	2007	Geometric	Pymol plugin, not available
FINDSITE[18]	2008	Template	Not available
PocketDepth[19]	2008	Geometric	Web server[20]

SitesIdentify[21]	2009	Geometry + Conservation	Not available
SiteMap[22]	2009	Geometric	Schrödinger software[23]
Fpocket[24]	2009	Geometric	Standalone application[25]
SiteHound[26,27]	2009	Energetic	Not available
ConCavity[28]	2009	Consensus (geometry + evolution)	Standalone application[29]
VICE[30]	2010	Geometric	Not available
3DLigandSite[31]	2010	Template	Not available
POCASA (Roll)[32]	2010	Geometric	Web server[33]
DoGSite[34]	2010	Geometric	Web server[35]
FunFOLD[36]	2011	Template	Web server[37]
MetaPocket 2.0[38]	2011	Consensus	Not available
MSPocket[39]	2011	Geometric	Not available
FTSite[40]	2012	Energetic	Web server[41]
LISE[42]	2012	Geometric	Not available
COFACTOR[43]	2012	Template	Web server[44]
COACH[45]	2013	Consensus	Web server[46]
eFindSite[47]	2013	Template	Standalone application[48]
GalaxySite[49]	2041	Template + docking	Web server[50]
LIBRA[51]	2015	Template	Standalone application[52]<br>Web server[53]
bSiteFinder[54]	2016	Template	Not available
ISMBLab-LIG[55]	2016	Machine learning	Not available
DeepSite[56]	2017	Deep learning	Web server[57]<br>https://www.playmolecule.com/deepsite/
P2Rank[58,59]	2018	Machine learning	Standalone application[60]<br>Web server[61]
GRaSP[62]	2020	Machine learning	Standalone application[63]

kalasanty[64]	2020	Deep learning	Standalone application[65]
DeepSurf[66]	2021	Deep learning	Standalone application[67]
PUResNet[68]	2021	Deep learning	Standalone application[69]
ScanNet[70]	2022	Deep learning	Standalone application[71]
DeepPocket[72]	2022	Deep learning	Standalone application[73]
SiteRadar[74]	2023	Deep learning	Upon request
SiteFerret[75]	2023	Geometric	Standalone application[76]
GrASP[77]	2024	Deep learning	Standalone application[78]
PUResNetV2.0[79]	2024	Deep learning	Standalone application[80]<br>Web server[81]

Table S5. Ligand names and corresponding complex PDB codes that were used to represent GPCR binding sites in Figure 1.

**GPCR Pocket**	**Ligand Name**	**PDB Code**
TM123_ext	BPTU	4XNV
TM1234567_ext	APD668	7XZ6
TM123567_ext	MS47134	7S8P
TM12367_ext	Ramatroban	6IIU
TM1237_ext	Taprenepag	7CX3
TM234567_ext	CHEMBL3894759	7EPE
TM2347_ext	MS47134	7S8P
TM3456_ext	Fasiglifam	8EJC

TM4567_ext	Evocalcet	7M3G
TM56_ext	MK-6892	8J6P
TM567_ext	2-chloranyl-~{N}-[2-methoxy-4-[(~{E})-pyridin-2-yldiazenyl]phenyl]benzamide	7P2L
TM23567_mid	CP-376395	4K5Y
TM34_mid	ZCZ011	7FEE
TM345_mid	(3~{S})-3-cyclopropyl-3-[2-[1-[2-[2,2-dimethylpropyl-(6-methylpyridin-2-yl)carbamoyl]-5-methoxy-phenyl]piperidin-4-yl]-1-benzofuran-6-yl]propanoic acid	5KW2
TM456_mid	(3S)-5,7-ditert-butyl-3-oxidanyl-3-(trifluoromethyl)-1-benzofuran-2-one	7C7Q
TM124678_int	CCR2-RA-[R]	5T1A
TM23678_int	(1R,2R)-N-[(2S,3S)-2-azanyl-3-methyl-pentyl]-N-[4-(4-propylphenyl)phenyl]-2-pyridin-2-yl-cyclopropane-1-carboxamide	7EJX
TM2678_int	PF-06372222	5VEW
TM34_int	Mevidalen	7LJC
TM6_int	N-Tert-butyl-6,7-dichloroquinoxalin-2-amine	7EVM

Table S6. Top-7 DCC and DVO success rates of ligand binding site prediction methods across all datasets.

**Method**	**Dataset**	**Top-7 DCC success rate, %**	**Top-7 DVO success rate, %**
Fpocket	GPCR	10	21
	Ion Channels	7	27
	PDBBind	36	42
ConCavity	**GPCR**	**33**	**49**
	Ion Channels	12	24
	PDBBind	40	85
**FTSite**	**GPCR**	8	**38**
	**Ion Channels**	14	**44**
	PDBBind	30	72
**P2Rank**	**GPCR**	**31**	23
	**Ion Channels**	**34**	22
	PDBBind	58	25
GRaSP	GPCR	0	3
	Ion Channels	0	2
	PDBBind	20	54
PUResNet	GPCR	15	28
	Ion Channels	2	7
	PDBBind	39	53
**DeepPocket**	**GPCR**	**31**	**62**
	**Ion Channels**	**17**	**71**
	PDBBind	56	86
**PUResNetV2.0**	**GPCR**	**28**	**56**
	**Ion Channels**	**22**	**53**
	PDBBind	64	86

SUPPORTING FIGURES

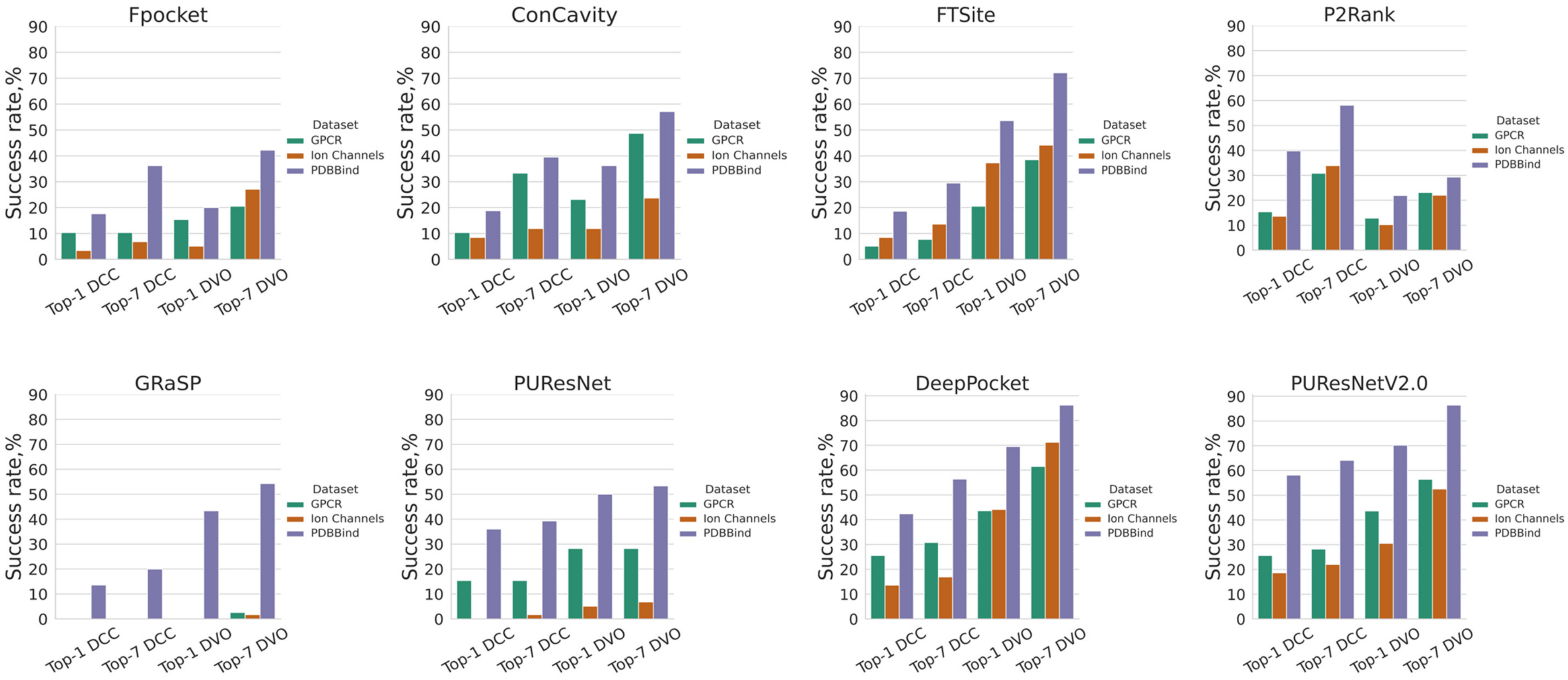

Figure S1. Performance comparison of various binding site prediction methods across used datasets in terms of top-1 and top-7 success rates in DCC (distance center to center) and DVO (discretized volume overlap) metrics.

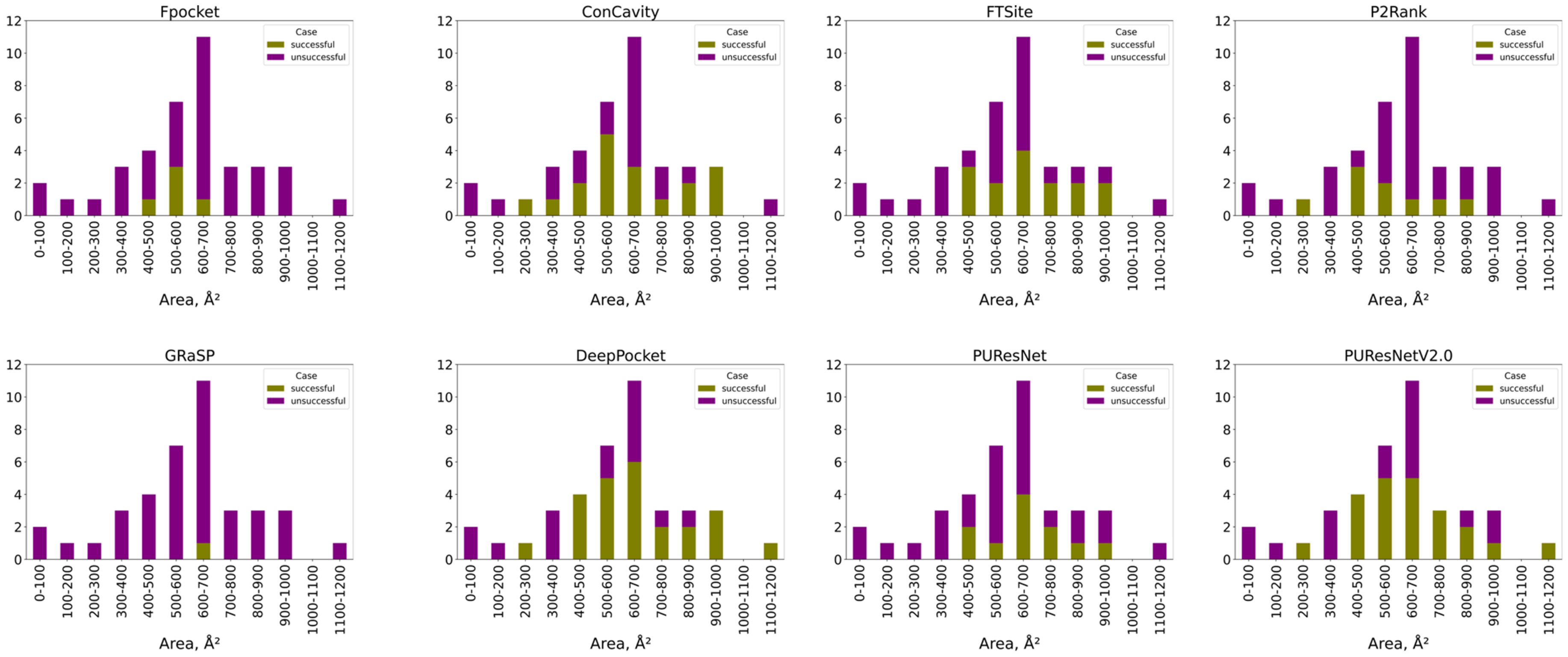

Figure S2. Distribution of successful and unsuccessful cases per binding site area range in GPCR dataset for every prediction method used in this study.

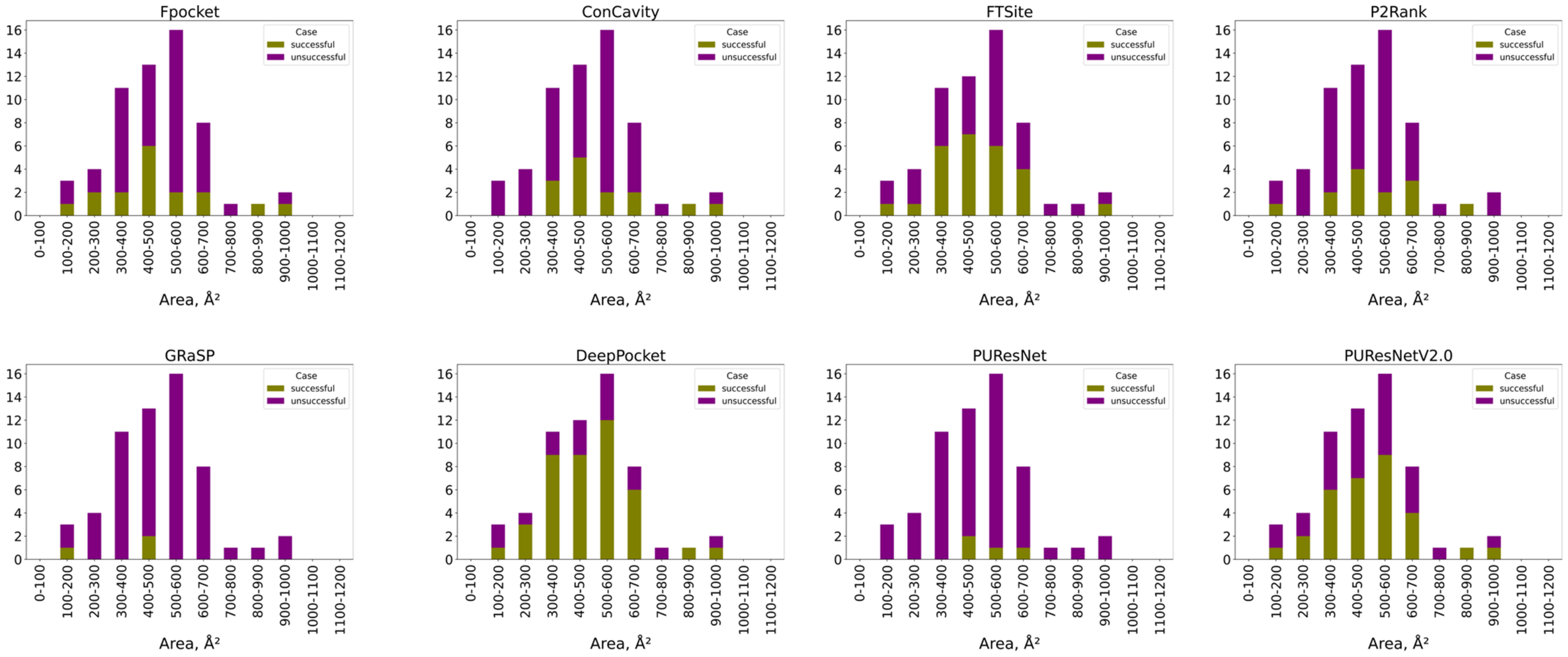

Figure S3. Distribution of successful and unsuccessful cases per binding site area range in ion channel dataset for every prediction method used in this study.

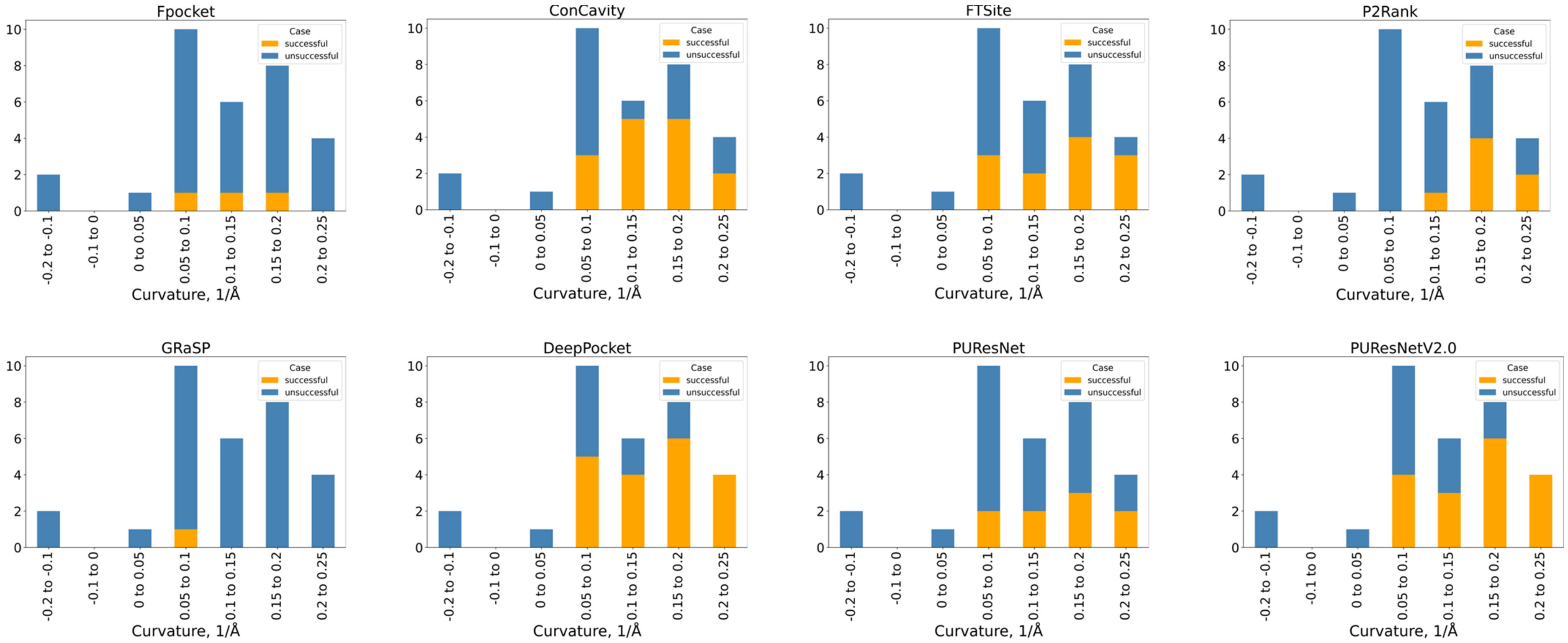

Figure S4. Distribution of successful and unsuccessful cases per binding site surface curvature in GPCR dataset for every prediction method used in this study.

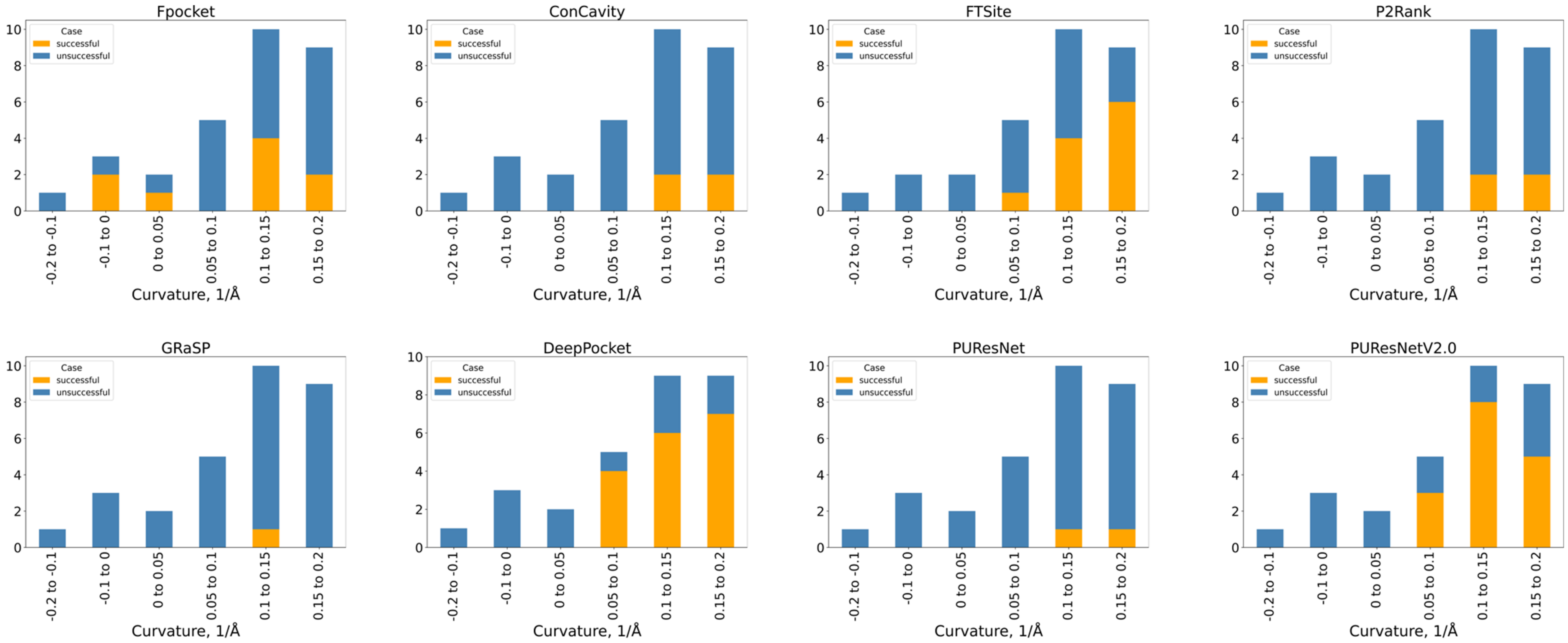

Figure S5. Distribution of successful and unsuccessful cases per binding site surface curvature in ion channel dataset for every prediction method used in this study.

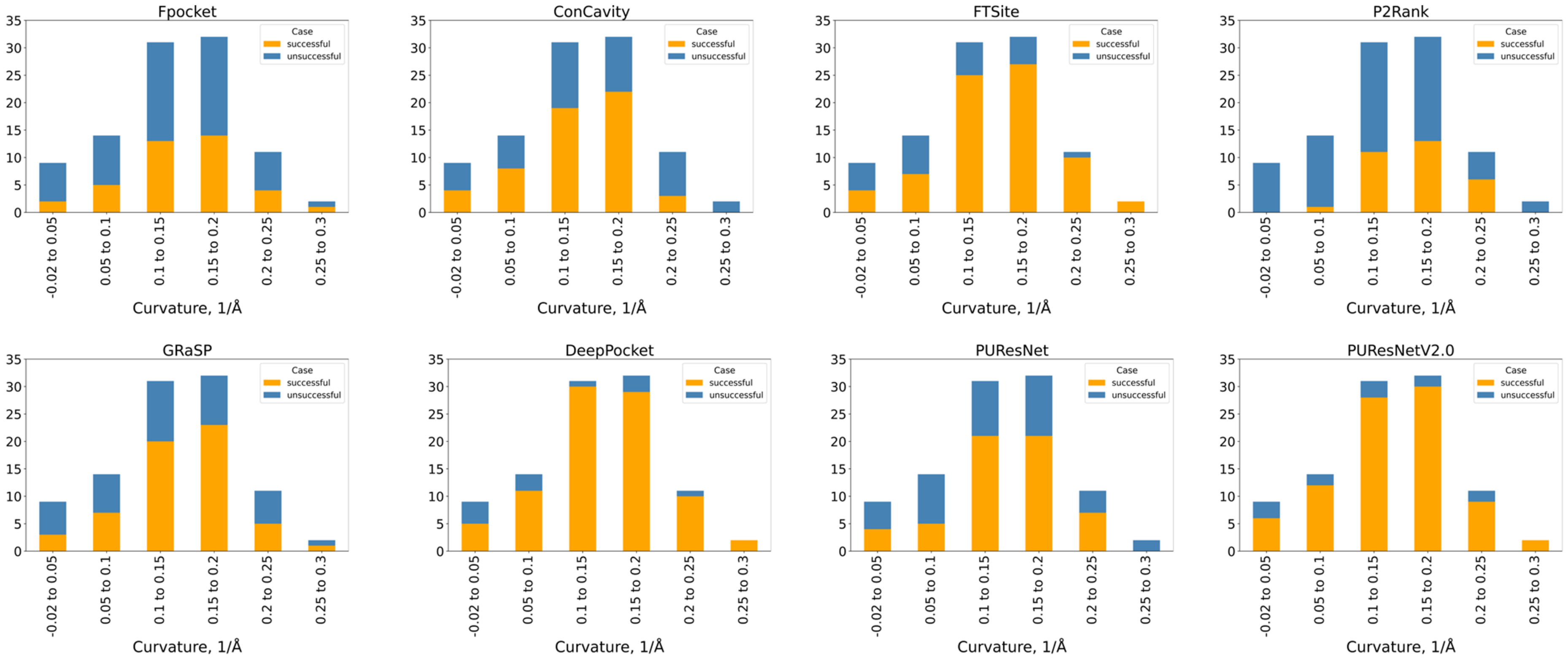

Figure S6. Distribution of successful and unsuccessful cases per binding site surface curvature in PDBBind dataset for every prediction method used in this study.

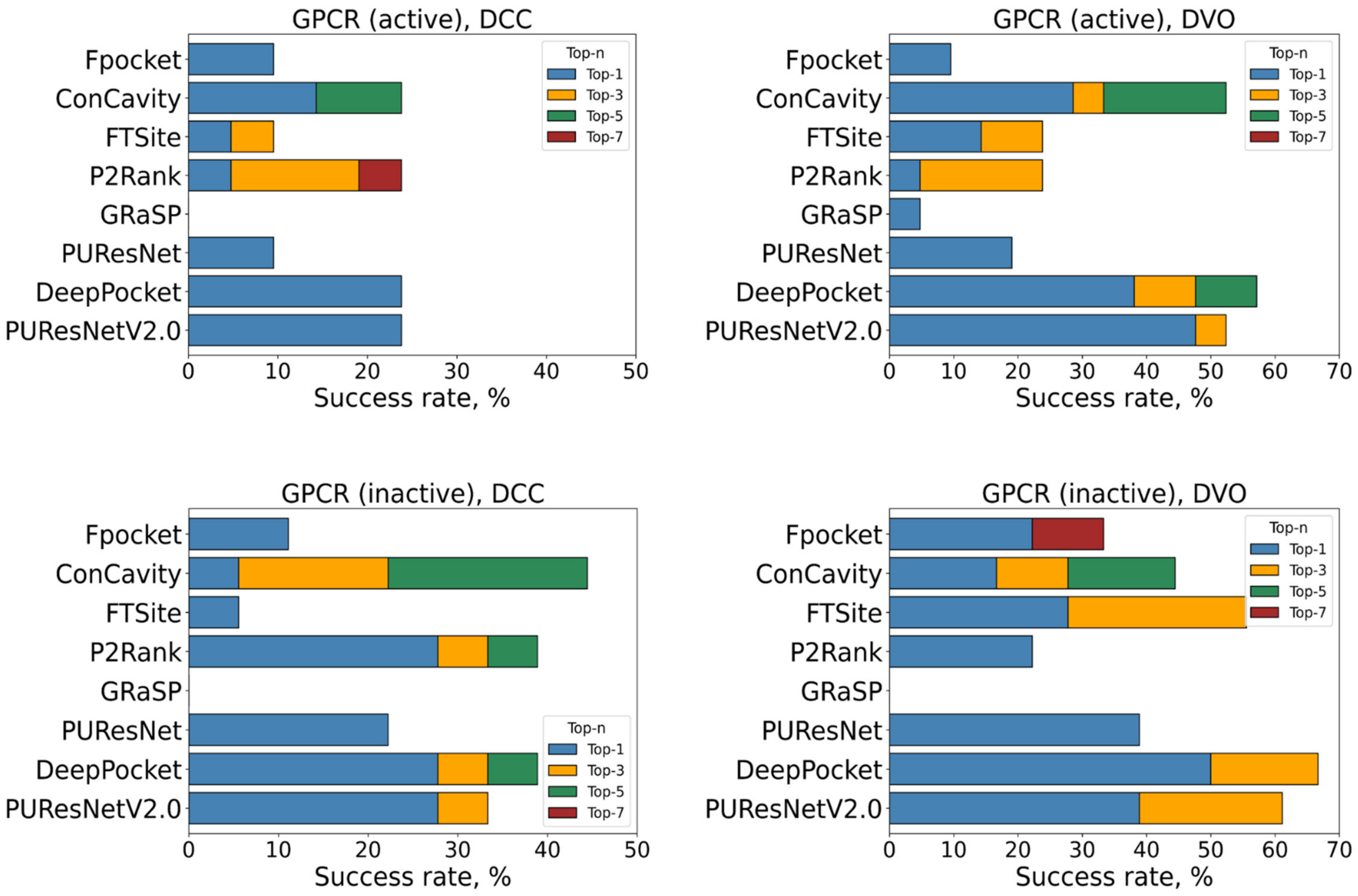

Figure S7. Performance comparison of ligand binding site prediction methods tested on active and inactive conformations of GPCRs. The left-hand side plots show the success rate according to DCC and the right-hand side plots compare the success rate according to DVO. X-axes represent top-n success rates with top-n being top-1, top-3, top-5, and top-7.

SUPPORTING REFERENCES

(1) Weininger D. SMILES, a Chemical Language and Information System. 1. Introduction to Methodology and Encoding Rules. *J. Chem. Inf. Comput. Sci* **1988**, *28* (1), 31–36. https://doi.org/https://doi.org/10.1021/ci00057a005.

(2) *Tsodikov Lab Software*. https://tsodikovlab.createuky.net/index_files/Surface_Racer.htm (accessed 2025-02-10).

(3) Richards, F. M. Areas, Volumes, Packing and Protein Structure. *Annu Rev Biophys* **1977**, No. 6, 151–176. https://doi.org/10.1146/annurev.bb.06.060177.001055.

(4) Levitt, D. G.; Banaszak, L. J. POCKET: A Computer Graphies Method for Identifying and Displaying Protein Cavities and Their Surrounding Amino Acids. *J Mol Graph* **1992**, *10* (4), 229–234. https://doi.org/10.1016/0263-7855(92)80074-N.

(5) Laskowski, R. A. SURFNET: A Program for Visualizing Molecular Surfaces, Cavities, and Intermolecular Interactions. *J Mol Graph* **1995**, *13* (5), 323–330. https://doi.org/10.1016/0263-7855(95)00073-9.

(6) Hendlich, M.; Rippmann, F.; Barnickel, G. LIGSITE: Automatic and Efficient Detection of Potential Small Molecule-Binding Sites in Proteins. *J Mol Graph Model* **1997**, *15* (6), 359–363. https://doi.org/10.1016/S1093-3263(98)00002-3.

(7) *Pocket Detection with LIGSITE*. https://enspara.readthedocs.io/en/latest/pocket-detection.html (accessed 2025-02-03).

(8) Brady, G. P.; Stouten, P. F. W. Fast Prediction and Visualization of Protein Binding Pockets with PASS. *J Comput Aided Mol Des* **2000**, *14* (4), 383–401. https://doi.org/10.1023/A:1008124202956.

(9) Tian, W.; Chen, C.; Lei, X.; Zhao, J.; Liang, J. CASTp 3.0: Computed Atlas of Surface Topography of Proteins. *Nucleic Acids Res* **2018**, *46* (W1), W363–W367. https://doi.org/10.1093/nar/gky473.

(10) *CASTp 3.0: Computed Atlas of Surface Topography of proteins*. http://sts.bioe.uic.edu/castp/calculation.html (accessed 2025-01-30).

(11) Laurie, A. T. R.; Jackson, R. M. Q-SiteFinder: An Energy-Based Method for the Prediction of Protein-Ligand Binding Sites. *Bioinformatics* **2005**, *21* (9), 1908–1916. https://doi.org/10.1093/bioinformatics/bti315.

(12) Huang, B.; Schroeder, M. LIGSITEcsc: Predicting Ligand Binding Sites Using the Connolly Surface and Degree of Conservation. *BMC Struct Biol* **2006**, *6*, 1–11. https://doi.org/10.1186/1472-6807-6-19.

(13) Soga, S.; Shirai, H.; Koborv, M.; Hirayama, N. Use of Amino Acid Composition to Predict Ligand-Binding Sites. *J Chem Inf Model* **2007**, *47* (2), 400–406. https://doi.org/10.1021/ci6002202.

(14) Soga, S.; Shirai, H.; Kobori, M.; Hirayama, N. Identification of the Draggable Concavity in Homology Models Using the PLB Index. *J Chem Inf Model* **2007**, *47* (6), 2287–2292. https://doi.org/10.1021/ci7002363.

(15) *MOE*. https://www.chemcomp.com/Products.htm (accessed 2024-10-10).

(16) López, G.; Valencia, A.; Tress, M. L. Firestar-Prediction of Functionally Important Residues Using Structural Templates and Alignment Reliability. *Nucleic Acids Res* **2007**, *35* (SUPPL.2), 573–577. https://doi.org/10.1093/nar/gkm297.

(17) Weisel, M.; Proschak, E.; Schneider, G. PocketPicker: Analysis of Ligand Binding-Sites with Shape Descriptors. *Chem Cent J* **2007**, *1* (1), 1–17. https://doi.org/10.1186/1752-153X-1-7.
(18) Skolnick, J.; Brylinski, M. FINDSITE: A Combined Evolution/Structure-Based Approach to Protein Function Prediction. *Brief Bioinform* **2009**, *10* (4), 378–391. https://doi.org/10.1093/bib/bbp017.
(19) Kalidas, Y.; Chandra, N. PocketDepth: A New Depth Based Algorithm for Identification of Ligand Binding Sites in Proteins. *J Struct Biol* **2008**, *161* (1), 31–42. https://doi.org/10.1016/j.jsb.2007.09.005.
(20) *PocketDepth*. http://proline.physics.iisc.ernet.in/pocketdepth/ (accessed 2025-01-31).
(21) Bray, T.; Chan, P.; Bougouffa, S.; Greaves, R.; Doig, A. J.; Warwicker, J. SitesIdentify: A Protein Functional Site Prediction Tool. *BMC Bioinformatics* **2009**, *10*, 1–12. https://doi.org/10.1186/1471-2105-10-379.
(22) Halgren, T. A. Identifying and Characterizing Binding Sites and Assessing Druggability. *J Chem Inf Model* **2009**, *49* (2), 377–389. https://doi.org/10.1021/ci800324m.
(23) Schrödinger Release 2021-4. *Desmond | Schrödinger*. D. E. Shaw Research. https://www.schrodinger.com/products/desmond (accessed 2023-02-20).
(24) Le Guilloux, V.; Schmidtke, P.; Tuffery, P. Fpocket: An Open Source Platform for Ligand Pocket Detection. *BMC Bioinformatics* **2009**, *10*, 1–11. https://doi.org/10.1186/1471-2105-10-168.
(25) *fpocket*. https://github.com/Discngine/fpocket (accessed 2025-01-30).
(26) Ghersi, D.; Sanchez, R. EasyMIFs and SiteHound: A Toolkit for the Identification of Ligand-Binding Sites in Protein Structures. *Bioinformatics* **2009**, *25* (23), 3185–3186. https://doi.org/10.1093/bioinformatics/btp562.
(27) Hernandez, M.; Ghersi, D.; Sanchez, R. SITEHOUND-Web: A Server for Ligand Binding Site Identification in Protein Structures. *Nucleic Acids Res* **2009**, *37* (SUPPL. 2), 413–416. https://doi.org/10.1093/nar/gkp281.
(28) Capra, J. A.; Laskowski, R. A.; Thornton, J. M.; Singh, M.; Funkhouser, T. A. Predicting Protein Ligand Binding Sites by Combining Evolutionary Sequence Conservation and 3D Structure. *PLoS Comput Biol* **2009**, *5* (12). https://doi.org/10.1371/journal.pcbi.1000585.
(29) *ConCavity*. https://compbio.cs.princeton.edu/concavity/ (accessed 2025-01-30).
(30) Tripathi, A.; Kellogg, G. E. A Novel and Efficient Tool for Locating and Characterizing Protein Cavities and Binding Sites. *Proteins: Structure, Function and Bioinformatics* **2010**, *78* (4), 825–842. https://doi.org/10.1002/prot.22608.
(31) Wass, M. N.; Kelley, L. A.; Sternberg, M. J. E. 3DLigandSite: Predicting Ligand-Binding Sites Using Similar Structures. *Nucleic Acids Res* **2010**, *38* (SUPPL. 2), 469–473. https://doi.org/10.1093/nar/gkq406.
(32) Yu, J.; Zhou, Y.; Tanaka, I.; Yao, M. Roll: A New Algorithm for the Detection of Protein Pockets and Cavities with a Rolling Probe Sphere. *Bioinformatics* **2009**, *26* (1), 46–52. https://doi.org/10.1093/bioinformatics/btp599.
(33) *POCASA1.1 on the web*. https://g6altair.sci.hokudai.ac.jp/g6/service/pocasa/ (accessed 2025-01-31).

(34) Volkamer, A.; Griewel, A.; Grombacher, T.; Rarey, M. Analyzing the Topology of Active Sites: On the Prediction of Pockets and Subpockets. *J Chem Inf Model* **2010**, *50* (11), 2041–2052. https://doi.org/10.1021/ci100241y.

(35) *DoGSiteScorer: Binding site prediction*. https://proteins.plus/help/dogsite (accessed 2025-01-31).

(36) Roche, D. B.; Tetchner, S. J.; Mcguffin, L. J. FunFOLD : An Improved Automated Method for the Prediction of Ligand Binding Residues Using 3D Models of Proteins FunFOLD : An Improved Automated Method for the Prediction of Ligand Binding Residues Using 3D Models of Proteins. *BMC Bioinformatics* **2011**, *12*. https://doi.org/10.1186/1471-2105-12-160.

(37) *The FunFOLD2 Protein-Ligand Binding Site Prediction Server*. https://www.reading.ac.uk/bioinf/FunFOLD/ (accessed 2025-01-31).

(38) Huang, B. Metapocket: A Meta Approach to Improve Protein Ligand Binding Site Prediction. *OMICS* **2009**, *13* (4), 325–330. https://doi.org/10.1089/omi.2009.0045.

(39) Zhu, H.; Pisabarro, M. T. MSPocket: An Orientation-Independent Algorithm for the Detection of Ligand Binding Pockets. *Bioinformatics* **2011**, *27* (3), 351–358. https://doi.org/10.1093/bioinformatics/btq672.

(40) Ngan, C. H.; Hall, D. R.; Zerbe, B.; Grove, L. E.; Kozakov, D.; Vajda, S. FtSite: High Accuracy Detection of Ligand Binding Sites on Unbound Protein Structures. *Bioinformatics* **2012**, *28* (2), 286–287. https://doi.org/10.1093/bioinformatics/btr651.

(41) *FTSite Server*. https://ftsite.bu.edu/ (accessed 2025-01-30).

(42) Xie, Z. R.; Liu, C. K.; Hsiao, F. C.; Yao, A.; Hwang, M. J. LISE: A Server Using Ligand-Interacting and Site-Enriched Protein Triangles for Prediction of Ligand-Binding Sites. *Nucleic Acids Res* **2013**, *41*, 292–296. https://doi.org/10.1093/nar/gkt300.

(43) Roy, A.; Yang, J.; Zhang, Y. COFACTOR: An Accurate Comparative Algorithm for Structure-Based Protein Function Annotation. *Nucleic Acids Res* **2012**, *40* (W1), 471–477. https://doi.org/10.1093/nar/gks372.

(44) *COFACTOR server*. https://zhanggroup.org/COFACTOR/ (accessed 2025-01-31).

(45) Yang, J.; Roy, A.; Zhang, Y. Protein-Ligand Binding Site Recognition Using Complementary Binding-Specific Substructure Comparison and Sequence Profile Alignment. *Bioinformatics* **2013**, *29* (20), 2588–2595. https://doi.org/10.1093/bioinformatics/btt447.

(46) *COACH: A meta-server based approach to protein-ligand binding site prediction*. https://zhanggroup.org/COACH/ (accessed 2025-01-31).

(47) Brylinski, M.; Feinstein, W. P. E FindSite : Improved Prediction of Ligand Binding Sites in Protein Models Using Meta-Threading , Machine Learning and Auxiliary Ligands. *J Comput Aided Mol Des* **2013**, *6* (27), 551–567. https://doi.org/10.1007/s10822-013-9663-5.

(48) *eFindSite*. https://www.brylinski.org/efindsite (accessed 2025-01-31).

(49) Heo, L.; Shin, W. H.; Lee, M. S.; Seok, C. GalaxySite: Ligand-Binding-Site Prediction by Using Molecular Docking. *Nucleic Acids Res* **2014**, *42* (W1), 210–214. https://doi.org/10.1093/nar/gku321.

(50) *GalaxyWEB*. https://galaxy.seoklab.org/cgi-bin/submit.cgi?type=SITE (accessed 2025-01-31).

(51) Viet Hung, L.; Caprari, S.; Bizai, M.; Toti, D.; Polticelli, F. LIBRA: LIgand Binding Site Recognition Application. *Bioinformatics* **2015**, *31* (24), 4020–4022. https://doi.org/10.1093/bioinformatics/btv489.

(52) *Theoretical Biology and Bioinformatics Laboratory*. https://www.computationalbiology.it/software.html (accessed 2025-01-31).
(53) *LIBRA WA*. http://biochimica3.bio.uniroma3.it/LIBRAWA/ (accessed 2025-01-31).
(54) Gao, J.; Zhang, Q.; Liu, M.; Zhu, L.; Wu, D.; Cao, Z.; Zhu, R. BSiteFinder , an Improved Protein - Binding Sites Prediction Server Based on Structural Alignment : More Accurate and Less Time - Consuming. *J Cheminform* **2016**, 1–10. https://doi.org/10.1186/s13321-016-0149-z.
(55) Jiménez, J.; Doerr, S.; Martínez-Rosell, G.; Rose, A. S.; De Fabritiis, G. DeepSite: Protein-Binding Site Predictor Using 3D-Convolutional Neural Networks. *Bioinformatics* **2017**, *33* (19), 3036–3042. https://doi.org/10.1093/bioinformatics/btx350.
(56) Jiménez, J.; Doerr, S.; Martínez-Rosell, G.; Rose, A. S.; De Fabritiis, G. DeepSite: Protein-Binding Site Predictor Using 3D-Convolutional Neural Networks. *Bioinformatics* **2017**, *33* (19), 3036–3042. https://doi.org/10.1093/bioinformatics/btx350.
(57) *DeepSite*. https://open.playmolecule.org/tools/deepsite (accessed 2025-02-03).
(58) Krivák, R.; Hoksza, D. P2RANK: Knowledge-Based Ligand Binding Site Prediction Using Aggregated Local Features. *Lecture Notes in Computer Science (including subseries Lecture Notes in Artificial Intelligence and Lecture Notes in Bioinformatics)* **2015**, *9199* (July 2015), 41–52. https://doi.org/10.1007/978-3-319-21233-3_4.
(59) Jendele, L.; Krivak, R.; Skoda, P.; Novotny, M.; Hoksza, D. PrankWeb: A Web Server for Ligand Binding Site Prediction and Visualization. *Nucleic Acids Res* **2019**, *47* (W1), W345–W349. https://doi.org/10.1093/nar/gkz424.
(60) *p2rank*. https://github.com/rdk/p2rank (accessed 2025-01-30).
(61) *PrankWeb*. https://prankweb.cz/ (accessed 2025-01-30).
(62) Santana, C. A.; de Silveira, S. A.; Moraes, J. P. A.; Izidoro, S. C.; de Melo-Minardi, R. C.; Ribeiro, A. J. M.; Tyzack, J. D.; Borkakoti, N.; Thornton, J. M. GRaSP: A Graph-Based Residue Neighborhood Strategy to Predict Binding Sites. *Bioinformatics* **2020**, *36* (26), I726–I734. https://doi.org/10.1093/bioinformatics/btaa805.
(63) *GRaSP*. https://github.com/charles-abreu/GRaSP (accessed 2025-01-30).
(64) Stepniewska-Dziubinska, M. M.; Zielenkiewicz, P.; Siedlecki, P. Improving Detection of Protein-Ligand Binding Sites with 3D Segmentation. *Sci Rep* **2020**, *10* (1), 1–9. https://doi.org/10.1038/s41598-020-61860-z.
(65) *kalasanty*. https://gitlab.com/cheminfIBB/kalasanty (accessed 2025-01-31).
(66) Mylonas, S. K.; Axenopoulos, A.; Daras, P. DeepSurf: A Surface-Based Deep Learning Approach for the Prediction of Ligand Binding Sites on Proteins. *Bioinformatics* **2021**, *37* (12), 1681–1690. https://doi.org/10.1093/bioinformatics/btab009.
(67) *DeepSurf*. https://github.com/stemylonas/DeepSurf (accessed 2025-01-31).
(68) Kandel, J.; Tayara, H.; Chong, K. T. PUResNet : Prediction of Protein - Ligand Binding Sites Using Deep Residual Neural Network. *J Cheminform* **2021**, 1–14. https://doi.org/10.1186/s13321-021-00547-7.
(69) *PUResNet*. https://github.com/jivankandel/PUResNet (accessed 2025-01-30).

(70) Tubiana, J.; Schneidman-duhovny, D.; Wolfson, H. J. ScanNet: An Interpretable Geometric Deep Learning Model for Structure-Based Protein Binding Site Prediction. *Nat Methods* **2022**, *19* (6), 730–739. https://doi.org/10.1038/s41592-022-01490-7.
(71) *ScanNet*. https://github.com/jertubiana/ScanNet (accessed 2025-01-31).
(72) Aggarwal, R.; Gupta, A.; Chelur, V.; Jawahar, C. V.; Priyakumar, U. D. DeepPocket: Ligand Binding Site Detection and Segmentation Using 3D Convolutional Neural Networks. *J Chem Inf Model* **2022**, *62* (21), 5069–5079. https://doi.org/10.1021/acs.jcim.1c00799.
(73) *DeepPocket*. https://github.com/devalab/DeepPocket (accessed 2025-01-30).
(74) Evteev, S. A.; Ereshchenko, A. V.; Ivanenkov, Y. A. SiteRadar: Utilizing Graph Machine Learning for Precise Mapping of Protein-Ligand-Binding Sites. *J Chem Inf Model* **2023**, *63* (4), 1124–1132. https://doi.org/10.1021/acs.jcim.2c01413.
(75) Gagliardi, L.; Rocchia, W. SiteFerret: Beyond Simple Pocket Identification in Proteins. *J Chem Theory Comput* **2023**, *19* (15), 5242–5259. https://doi.org/10.1021/acs.jctc.2c01306.
(76) *SiteFerret*. https://github.com/concept-lab/SiteFerret (accessed 2025-01-31).
(77) Smith, Z.; Strobel, M.; Vani, B. P.; Tiwary, P. Graph Attention Site Prediction (GrASP): Identifying Druggable Binding Sites Using Graph Neural Networks with Attention. *J Chem Inf Model* **2023**. https://doi.org/10.1021/acs.jcim.3c01698.
(78) *GrASP*. ttps://github.com/tiwarylab/GrASP (accessed 2025-01-31).
(79) Jeevan, K.; Palistha, S.; Tayara, H.; Chong, K. T. PUResNetV2.0: A Deep Learning Model Leveraging Sparse Representation for Improved Ligand Binding Site Prediction. *J Cheminform* **2024**, *16* (1), 1–16. https://doi.org/10.1186/s13321-024-00865-6.
(80) *PUResNetV2.0*. https://github.com/jivankandel/PUResNetV2.0 (accessed 2025-01-30).
(81) *PURESNET*. https://nsclbio.jbnu.ac.kr/tools/jmol (accessed 2025-01-30).
(82) Qian, Y.; Wang, J.; Yang, L.; Liu, Y.; Wang, L.; Liu, W.; Lin, Y.; Yang, H.; Ma, L.; Ye, S.; Wu, S.; Qiao, A. Activation and Signaling Mechanism Revealed by GPR119-Gs Complex Structures. *Nat Commun* **2022**, *13* (1). https://doi.org/10.1038/s41467-022-34696-6.
(83) Mao, C.; Gao, M.; Zang, S. K.; Zhu, Y.; Shen, D. D.; Chen, L. N.; Yang, L.; Wang, Z.; Zhang, H.; Wang, W. W.; Shen, Q.; Lu, Y.; Ma, X.; Zhang, Y. Orthosteric and Allosteric Modulation of Human HCAR2 Signaling Complex. *Nat Commun* **2023**, *14* (1). https://doi.org/10.1038/s41467-023-43537-z.
(84) Yang, Y.; Wei, M.; Chen, L. Structural Identification of Riluzole-Binding Site on Human TRPC5. *Cell Discovery*. Springer Nature December 1, 2022. https://doi.org/10.1038/s41421-022-00410-5.
(85) Nannan, S.; Wenxuan, Z.; Heng, Z.; Lingyi, X.; Yitian, J.; Xiaoying, C.; Cheng, Z.; Qinrui, W.; Xinyan, W.; Shaowei, L.; Han, W.; Jiangtao, G.; Fan, Y. Structural Mechanisms of TRPV2 Modulation by Endogenous and Exogenous Ligands. *Nat Chem Biol* **2023**, *19* (1), 72–80. https://doi.org/10.1038/s41589-022-01139-8.
